# PLK1 and AURKB phosphorylate survivin differentially to affect proliferation in racially distinct triple-negative breast cancer

**DOI:** 10.1038/s41419-022-05539-5

**Published:** 2023-01-10

**Authors:** Chakravarthy Garlapati, Shriya Joshi, Shristi Bhattarai, Jayashree Krishnamurthy, Ravi Chakra Turaga, Thi Nguyen, Xiaoxian Li, Ritu Aneja

**Affiliations:** 1 Alkermes Inc, Waltham, MA 02451 USA; 2 Department of Biology, Georgia State University, Atlanta, GA 30303 USA; 3 Caris Life Sciences, Tempe, AZ 85282 USA; 4 Department of Pathology & Laboratory Medicine, Emory University School of Medicine, Atlanta, GA 30322 USA; 5 School of Health Professions, University of Alabama at Birmingham, Birmingham, AL 35294 USA

**Keywords:** Phosphorylation, Breast cancer

## Abstract

Protein diversity due to alternative mRNA splicing or post-translational modifications (PTMs) plays a vital role in various cellular functions. The mitotic kinases polo-like kinase 1 (PLK1) and Aurora B (AURKB) phosphorylate survivin, an inhibitor of apoptosis (IAP) family member, thereby regulating cell proliferation. PLK1, AURKB, and survivin are overexpressed in triple-negative breast cancer (TNBC), an aggressive breast cancer subtype. TNBC is associated with high proliferative capacity, high rates of distant metastasis, and treatment resistance. The proliferation-promoting protein survivin and its activating kinases, PLK1 and AURKB, are overexpressed in TNBC. In this study, we investigated the role of survivin phosphorylation in racial disparities in TNBC cell proliferation. Analysis of TCGA TNBC data revealed higher expression levels of *PLK1* (*P* = 0.026) and *AURKB* (*P* = 0.045) in African Americans (AAs; *n* = 41) than in European Americans (EAs; *n* = 86). In contrast, no significant racial differences in survivin mRNA or protein levels were observed. AA TNBC cells exhibited higher p-survivin levels than EA TNBC cells. Survivin silencing using small interfering RNAs significantly attenuated cell proliferation and cell cycle progression in AA TNBC cells, but not in EA TNBC cells. In addition, PLK1 and AURKB inhibition with volasertib and barasertib significantly inhibited the growth of AA TNBC xenografts, but not of EA TNBC tumors. These data suggest that inhibition of PLK1 and AURKB suppresses cell proliferation and tumor growth, specifically in AA TNBC. These findings suggest that targeting survivin phosphorylation may be a viable therapeutic option for AA patients with TNBC.

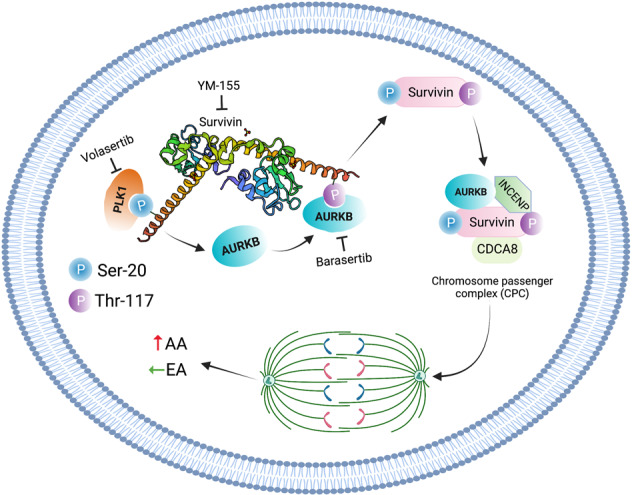

## Introduction

The breast cancer (BC) subtype triple-negative BC (TNBC) accounts for 15%–20% of all BC cases in the US [1]. TNBCs lack expression of estrogen receptor (ER), progesterone receptor (PR), and human epidermal growth factor receptor (HER2), all of which are therapeutic targets. Currently, there are no FDA-approved targeted therapies for patients with TNBC, whose survival outcomes are dismal [2–5]. Women of African descent are twice as likely to develop TNBC as women of European descent [6]. AA patients with TNBC show higher cell proliferation than their EA counterparts, contributing to the aggressive disease course and poor prognosis observed in AAs with TNBC [7–9]. However, the molecular mechanisms underlying high TNBC cell proliferation in AAs remain unknown.

The molecular basis of disparities in TNBC may not be restricted to intrinsic gene expression-based biological differences. Protein diversity stemming from alternative mRNA splicing or post-translational modifications (PTM) modulates cellular functions and protein–protein and protein–lipid crosstalk [10]. PTMs regulate various biological processes, including cell proliferation [11], cell differentiation [11], and carcinogenesis [12]. Advances in proteomics have fueled investigations into the role of PTMs and have revealed that PTMs generate a complex combinatorial code that regulates gene expression and protein function. Proteomic studies have also revealed profound deregulation of PTMs in various cancer types [13]. Phosphorylation, acetylation, lipidation, SUMOylation, methylation, and glycosylation are PTMs that are highly relevant in cancer and rewire various oncogenic signaling pathways. A better understanding of PTM profiles in cancer may provide next-generation biomarkers for improved disease prognosis and uncover a protein network amenable to therapeutic targeting in a spatiotemporal manner.

Cell division requires chromosome segregation, which is orchestrated by the interaction between spindle microtubules and the centromere. Accurate attachment of spindle microtubules to the kinetochore requires the chromosomal passenger complex (CPC), which comprises Aurora kinase B (AURKB), Borealin, INCENP, and survivin, among other proteins [14]. Chu et al. [15]. have demonstrated that AURKB activation relies on other mitotic kinases, including polo-like kinase 1 (PLK1). They have also shown that PLK1 phosphorylates survivin at Ser-20. In turn, survivin activates AURKB, thereby promoting cell division. These data support the hypothesis that PTMs in survivin regulates cell proliferation.

Survivin is involved in various processes that regulate cancer progression, including cell proliferation, apoptosis, angiogenesis, and drug resistance. Survivin is of significant interest as a therapeutic target because of its high expression in cancerous tissues and cell lines. Elevated survivin levels have also been associated with poor prognosis in patients with TNBC [16]. However, little is known about the role of survivin in racially diverse TNBC populations. Our findings suggest that phosphorylation of survivin (S20 and T117) by two mitotic kinases, PLK1 and AURKB, is essential for cell proliferation in AA patients with TNBC and could serve as a viable therapeutic target for AAs with TNBC. This study provides a rationale for the development of combinatorial therapies targeting PLK1 and AURKB in AA patients with TNBC.

## Methods

All reagents and antibodies used are listed in Table 1.

**Table 1:** List of reagents and antibodies.

No.	Name of reagent/antibody	Dilution/concentration	Catalog no.	Company
1	Lipofectamine RNAiMAX	2%	13,778,075	ThermoFisher Scientific
2	Signal silence Survivin siRNA	100 nM final concentration	6351	Cell Signaling Technology
3	Anti-survivin antibody	1:1000 (WB) 1:100 (IHC)	2808S	Cell Signaling Technology
4	RNAeasy mini kit		74,104	Qiagen
5	iScript cDNA synthesis kit	1×	1,708,891	BioRad
6	SsoAdvanced Universal SYBR Green Supermix	1×	1,725,271	BioRad
7	Protease inhibitor cocktail	1×	P8340	Sigma Aldrich
8	Anti-AURKB antibody	1:500	NBP261493	Novus Biologicals
9	Anti-p-survivin (S20) antibody	1:1000 (WB) 1:100 (IHC-P)	NB11092717	Novus Biologicals
10	Anti-p-survivin (T117) antibody	1:1000 (WB) 1:100 (IHC-P)	MBS003339	MyBioSource
11	Anti-β-actin antibody	1:1000 (WB)	SC-47778	Santa Cruz Biotechnology
12	Goat anti-mouse HRP antibody	1:8000 or 1:10,000	SC-2005	Santa Cruz Biotechnology
13	Goat anti-rabbit HRP antibody	1:8000 or 1:10,000	4050-05	Southern Biotech
14	ECL kit		32106	Thermo Fisher Scientific
15	BrdU cell proliferation kit		2750	EMD Millipore
16	BrdU antibody	1:1000	ab152095	Abcam
17	α tubulin antibody	1:400 (IF)	T9026	Sigma Aldrich
18	Volasertib	15 mg/kg	HY-12137	MedChem Express
19	Barasertib-HQPA		HY-10126	MedChem Express
20	1× phosphate buffer saline		MT21040CV	Corning
21	Propidium iodide	50 µg/mL	P4170-10MG	MilliporeSigma
22	RNase	100 μg/mL	EN0531	Thermo Fisher Scientific
23	37% paraformaldehyde		252549-500 ML	MilliporeSigma
24	Crystal violet	1×	C0775-25G	MilliporeSigma
25	Diva 10× antigen retrieval buffer	1×	DV2004LX	Biocare Medical
26	Anti-Ki-67 antibody	1:100	CRM325C	Biocare Medical
27	pHH3 antibody	1:500	3130	Biocare Medical
28	Anti-PLK1 antibody	1:50	4513 S	Cell Signaling Technology
29	Rabbit HRP antibody	1×	RHRP520L	Biocare Medical
30	Mouse HRP antibody	1×	MHRP520L	Biocare Medical
31	Nude (nu/nu) mice		002019	Jackson Laboratories
32	Barasertib	100 mg/kg	HY-10127	MedChem Express
33	YM-155	10 mg/kg	T2111-SB200	TargetMol
34	ALZET Micro-osmotic Pumps		Model 1004	ALZET
35	Maxi prep kit		12165	Qiagen
36	Lipofectamine LTX Plus	LTX (7.5 μl/well) Plus-reagent (5 μl/2.5 μg plasmid/well) in a 6-well plate	15338030	Thermo Fisher Scientific
37	Anti-Flag-M2 magnetic beads	25 μl/reaction	M8823	MilliporeSigma
38	Anti-Borealin antibody	1:500 (WB)	sc-376635	Santa Cruz Biotechnology
39	Anti-INCENP antibody	1:500 (WB)	ab12183	Abcam
40	Corning BioCoat Matrigel Invasion Chambers with 8.0 µm PET Membrane		354480	Corning
41	PLK1 siRNA,	100 nM final conc	6292, AM5133	Cell Signaling Technology, Thermo Fisher Scientific
42	AURKB siRNA	100 nM final conc	L00332600-0005, AM16708	Horizon Discovery Thermo Fisher Scientific
43	DAB Chromogen Kit		DB801	Biocare Medical
44	PLK1 (NM_005030) Human Untagged Clone		SC110978	OriGene
45	Aurora B (AURKB) (NM_004217) Human Tagged ORF Clone		RC210288	OriGene

### Cell culture

The TNBC cell lines MDA-MB-468, HCC1806, and MDA-MB-157 (AA); MDA-MB-231, HCC1143, and HCC1937 (EA); as well as HEK-293 cells, were purchased from ATCC. All cell lines were cultured in the appropriate medium containing 10% fetal bovine serum and 1% antibiotics and were maintained at 37 °C in a 5% CO_2_ atmosphere.

### Cell transfection

Cells at 70–80% confluency were trypsinized and seeded for transfection with siRNAs or PLK1 and AURKB overexpression (OE) plasmids; transfections were performed using RNAimax or Lipofectamine LTX plus according to the manufacturer’s guidelines. Knockdown (KD) or OE efficiency was assessed by immunoblotting 36 h after transfection. Cells with >90% KD efficiency were selected for further analyses.

### Real-time polymerase chain reaction

mRNA from TNBC cells was extracted using the RNeasy kit as described previously [17, 18]. The iScript cDNA synthesis kit was used to generate cDNA per the manufacturer’s instructions. Polymerase chain reaction was performed using SsoAdvanced Universal SYBR Green Supermix, and β-actin was used as the reference gene. The following primers were used:

β-actin: forward, 5′-CTGGAACGGTGAAGGTGACA-3′; reverse 5′-AAGGGACTTCCTGTAACAATGCA-3′

PLK1: forward, 5′-CACAGTTTCGAGGTGGATGT-3′; reverse 5′-ATCCGGAGGTAGGTCTCTTT-3′

AURKB: forward, 5′-GGAGTTGGCAGATGCTCTAAT-3′; reverse 5′-CAATCTTCAGCTCTCCCTTGAG-3′

BIRC5: forward, 5′-CTTGGCCCAGTGTTTCTTCT-3′; reverse 5′-CCGCAGTTTCCTCAAATTCTTTC-3′

### Immunoblotting

Immunoblotting was performed as previously described [19]. Primary antibodies against AURKB, survivin, p-survivin (S20), p-survivin (T117), and β-actin were used. Goat anti-mouse or goat anti-rabbit IgG horseradish peroxidase (HRP) secondary antibodies were used, and the signal was visualized using an ECL kit. Protein levels were quantified using ImageJ and were normalized to their respective β-actin controls.

### Cell proliferation assay and immunofluorescence

Cell proliferation was evaluated using BrdU. Survivin KD and control cells were seeded (5 × 10^3^ cells per well) and incubated with BrdU for 4 h. BrdU incorporation was measured spectrophotometrically at 450 nm using TMB substrate. For immunofluorescence (IF) assays, cells were incubated with BrdU for 24 h at 37 °C. Cells were fixed in formaldehyde and were subjected to acid hydrolysis followed by neutralization with borate buffer. After blocking, cells were incubated with primary antibodies (a cocktail of BrdU and α-tubulin antibodies). Cells were incubated with a rabbit and mouse fluorescent secondary antibody cocktail at 37 °C for 40–45 min. Nuclei were counterstained with Hoechst, and coverslips were mounted with ProLong Gold Antifade. Images were acquired using a confocal microscope (LSM 700; ZEISS) and analyzed using ImageJ. IF for survivin was performed as described above but without BrdU incubation and acid hydrolysis, as previously described [20].

### Cell cycle analysis

Cells were treated with different agents for 48 h. After incubation, cells were fixed in 70% ice-cold ethanol for 30 min, followed by centrifugation. The cell pellet was dissolved in RNase A (100 µg/mL) and was incubated at 37 °C for 30 min. After incubation, 200 µL propidium iodide (PI; 50 µg/mL) was added to the cells; 200 µL PBS was added to unstained cells. Cell cycle analysis was performed using a BD Fortessa flow cytometer, and the percentage of cells in each cell cycle phase was determined using FlowJo.

### Boyden chamber invasion assay

Boyden chamber assay was performed as described previously [21]. Survivin KD and control cells (~2 × 10^5^) were mixed with serum-free medium and seeded on inserts with 8 µM pores in 24-well plates. The plates were incubated for 12–18 h at 37 °C. Boyden chambers were fixed with 3.7% paraformaldehyde and stained with 4% crystal violet. Five different fields for every sample were observed, purple colonies were counted independently by two observers, and the mean colony count was determined. Images were acquired using ToupView.

### Scratch wound migration assay

Survivin KD and control cells were seeded, and a wound was scratched gently. Images were obtained from six different fields using a ZEISS Primovert inverted phase-contrast microscope. Images were acquired at 0 and 24 h, and wound closure and migration efficiency were analyzed using ImageJ and Adobe Photoshop [21].

### Immunohistochemistry

To determine the mitosis score and survivin nuclear *H* score, we used tissue samples from TNBC patients from Emory (AA: *n* = 76, EA: *n* = 24) and Dekalb (AA: *n* = 32, EA: *n* = 16) hospitals. Formalin-fixed paraffin-embedded (FFPE) tissue sections (5 µm) were deparaffinized and rehydrated in serial ethanol solutions as previously described [17]. Antigen retrieval was achieved by incubation in Diva 1× (pH 6.0) buffer in a pressure cooker for 10 min at high pressure. Ki-67, pHH3, PLK1, AURKB, survivin, p-survivin (S20), and p-survivin (T117) were immunostained. Mach2 mouse/rabbit HRP antibody was used for enzymatic detection of primary antibodies. Biomarkers were reviewed and scored by two independent pathologists. The intensity of staining (none = 0, low = 1, moderate = 2, high = 3) and the percentage of positive cells was determined, and the scores of the two pathologists were averaged. Weighted indexes were determined by multiplying the staining intensity score by the percentage of positive cells.

### Xenograft animal model

Nude female mice were used, and all protocols adhered to the guidelines of the Institutional Animal Care and Use Committee guidelines. To determine the number of animals required for the study, we performed power analysis using GraphPad Prism 9 software [22]. HCC1806 (AA) and MDA-MB-231 (EA) cells were subcutaneously injected into the right flank of mice (4 × 10^6^ cells per flank). When tumors reached 100 mm^3^, mice were divided into four groups: vehicle, volasertib (15 mg/kg), barasertib (100 mg/kg), volasertib plus barasertib(15 mg/kg + 100 mg/kg), and YM155 (*n* = 12 for each treatment and race group). All drugs except YM-155 were administered intraperitoneally twice weekly for up to 28 days. For YM-155, Alzet micro-osmotic pumps were surgically implanted subcutaneously into tumor-bearing mice. YM-155 (10 mg/kg, 0.11 µL per hour) 4-day continuous infusion per week was administered through micro-osmotic pumps for 2 weeks. Tumor growth was measured once per week using Vernier calipers, and body weight was recorded for up to 4 weeks. Tumor volume was calculated as follows: length × (width)^2^ ÷ 2. All mice were euthanized at the end of the experiment, and tumors were collected and fixed in 10% formalin. FFPE blocks were prepared, and tissue sections (5 µm) were stained with H&E to confirm the tumor area. Six mice per group were monitored for survival for 90 days after xenografting. Fresh-frozen tumor sections from euthanized mice were used for lysate preparation using a BeadBlaster homogenizer.

### Immunoprecipitation

NCBI BLAST and SnapGene were used to design survivin phospho-mutant and wild-type (WT) plasmids. The phospho-mutant plasmids phospho-mimic (E- glutamic acid; S20, T117 single and double mutant) and phospho-stop (A-alanine; S20, T117 single and double mutants) were purchased from GenScript. Plasmids. Lipofectamine LTX PLUS was used to transfect HEK293 cells with the plasmids. Cell lysates were prepared after 48 h, and lysates (1 mg/mL protein) were used for immunoprecipitation (IP) analysis using anti-Flag-M2 magnetic beads. Proteins were eluted using glycine HCl (pH 2.5–3) and neutralized using 0.5 M Tris 1.5 M NaCl (pH 8). Bound proteins and input controls were used for immunoblotting.

### Statistical analysis

All experiments were performed in triplicate, and data were used to calculate statistical significance using two-tailed unpaired Student’s *t*-test with Welch’s correction, unpaired nonparametric Mann–Whitney or Kolmogorov–Smirnov test, or one- or two-way analysis of variance (ANOVA) with Tukey’s test for multiple comparisons. Survival data were analyzed using the Mantel–Cox test, and Pearson’s coefficient was used to assess correlations among variables. Data were expressed as mean ± standard error of the mean. Statistical analyses were performed using GraphPad Prism version 9.

## Results

### PLK1 and AURKB levels are higher in AA than in EA patients with TNBC

**Fig. 1:**
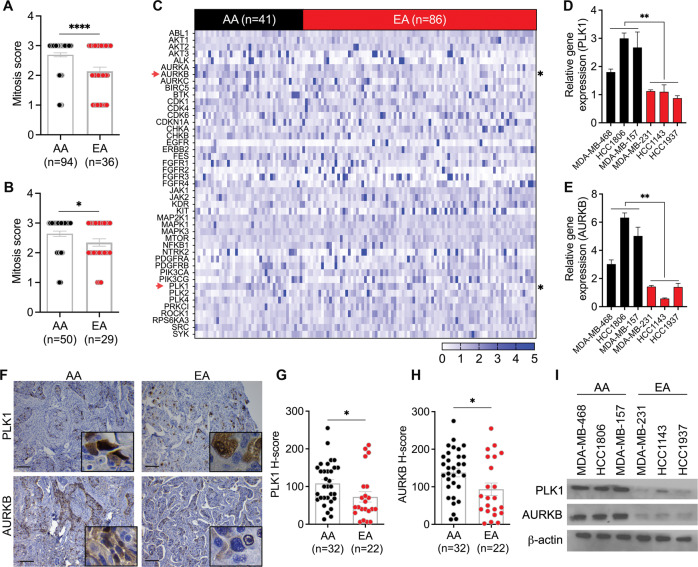
PLK1 and AURKB levels in TNBC are higher in AAs than in EAs. Bar graphs showing mitosis scores in the Emory (**A**) and Dekalb (**B**) cohorts. **C** Heatmap showing the expression levels of various kinases in the TCGA BC dataset. **D**, E Bar graphs showing the expression levels of PLK1 (**D**) and AURKB (**E**) in AA (*n* *=* 3) and EA (*n* *=* 3) TNBC cell lines. **F**–**H** Representative IHC images of PLK1 and AURKB (**F**) and quantification bar graphs showing PLK1 (**G**) and AURKB (**H**) levels in grade- and stage-matched AA and EA patients with TNBC (Dekalb cohort). **I** Immunoblot showing PLK1 and AURKB protein levels in AA and EA TNBC cell lines (*n* *=* 3 each). FPKM fragments per kilobase of transcript per million mapped reads. Bars indicate mean ± SEM. Unpaired two-tailed Student’s *t*-test with Welch’s correction was used to determine statistical significance (*P *<* 0.05, ***P *<* 0.0005, ns = non-significant). The scale bar represents 100 µm.

High cancer cell proliferation in AAs is believed to contribute to the more aggressive TNBC course in AAs than in EAs. TNBC mortality is higher in AA women than in women of European descent. We and others [6] have validated these results in various cohorts (Supplementary Fig. 1A, B). Most TNBCs exhibit marked nuclear pleomorphism and numerous mitoses [17]. In this study, we evaluated the mitotic scores in EA and AA patients with TNBC and found a higher mitotic score in TNBC tissues in AAs than in EAs (Fig. 1A, B). Mitosis is regulated by various protein kinases [23]. Based on the high mitotic score and proliferative index in AA patients with TNBC, we evaluated the expression of various mitotic and cyclin-dependent kinases in racially diverse patients. In silico analysis of the publicly available TCGA BC dataset (filtered for TNBC; AA: *n* = 41, EA: *n* = 86) revealed significantly higher expression levels of the mitotic kinases *PLK1* (*P* = 0.026) and *AURKB* (*P* = 0.045) in AA patients than in EA patients (Fig. 1C; Supplementary Fig. 2A, B; Table 2). Immunohistochemical staining of FFPE TNBC tissue samples (Dekalb cohort; AA: *n* = 32, EA: *n* = 22; Fig. 1F) revealed higher *H* scores for PLK1 and AURKB in AAs than in EAs (Fig. 1G, H). We also assessed the mRNA and protein levels of PLK1 and AURKB in selected AA (*n* = 3) and EA (*n* = 3) TNBC cell lines. *PLK1* and *AURKB* mRNA and protein levels were higher in AA TNBC cells than in EA TNBC cells (Fig. 1D, E, I). PLK1 and AURKB are involved in multiple mitotic events, including centrosome maturation, kinetochore spindle attachment, and cytokinesis. PLK1 and AURKB often phosphorylate different components involved in the same mitotic process [23]. Therefore, the high mitotic score and cell proliferation observed in AAs with TNBC may be attributed to the high expression levels of PLK1 and AURKB.

**Table 2:** Expression of various kinases in AA and EA patients with TNBC (TCGA dataset).

Sr. no	Gene	Below threshold	*P*-value
1	ABL1	No	0.376
2	AKT1	No	0.153
3	AKT2	No	0.327
4	AKT3	No	0.151
5	ALK	No	0.701
6	AURKA	No	0.732
7	AURKB	Yes	0.001
8	AURKC	No	0.681
9	BTK	No	0.307
10	CDK1	No	0.914
11	CDKN1A	No	0.919
12	CHKA	No	0.296
13	CHKB	No	0.133
14	SRC	No	0.243
15	EGFR	No	0.198
16	ERBB2	No	0.413
17	FES	No	0.023
18	FGFR1	No	0.576
19	FGFR2	No	0.265
20	FGFR3	No	0.752
21	FGFR4	No	0.943
22	PIK3CA	No	0.13
23	JAK2	No	0.448
24	KIT	No	0.126
25	MAPK1	No	0.225
26	MAPK3	No	0.28
27	MAP2K1	No	0.868
28	MTOR	No	0.015
29	NFKB1	No	0.046
30	PDGFRA	No	0.369
31	PDGFRB	No	0.549
32	PIK3CA	No	0.13
33	PRKCI	No	0.095
34	PLK1	Yes	0.026
35	PLK2	No	0.528
36	PLK3	No	0.178
37	PLK4	No	0.069
38	PIK3CG	No	0.591
39	ROCK1	No	0.027
40	RPS6KA3	No	0.126
41	SYK	No	0.233
42	SRC	No	0.243
43	NTRK2	No	0.423
44	KDR	No	0.047

### Survivin expression, localization, and phosphorylation in AAs and EAs with TNBC

**Fig. 2:**
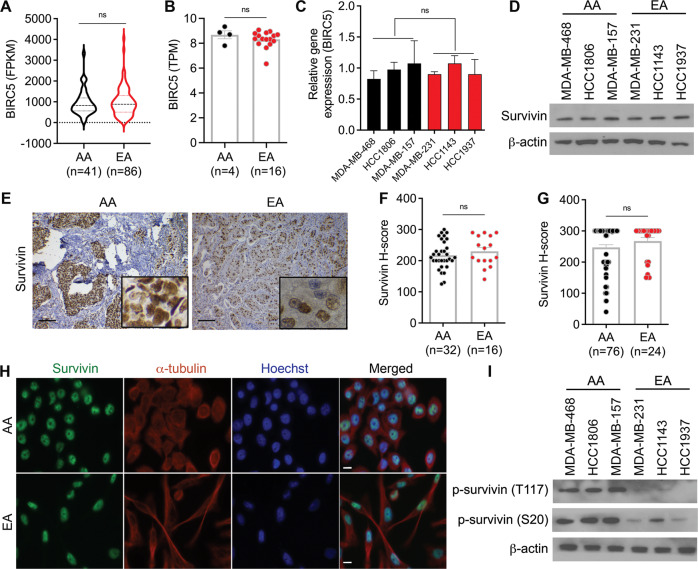
Survivin expression, localization, and phosphorylation in AAs and EAs with TNBC. **A** *BIRC5* expression level in AA and EA patients with TNBC (TCGA dataset). **B** Relative *BIRC5* expression in AA and EA TNBC cell lines from Neve et al. (2006) (**B**) and our in-house TNBC cell lines (**C**) (*n* = 3 each). **D** Immunoblot showing survivin levels in AA (*n* = 3) and EA (*n* = 3) TNBC cell lines. **E**–**G** Representative IHC images (**E**) and bar graphs (**F**, **G**) showing survivin *H* scores in AA and EA patients with TNBC in the Dekalb (**F**) and Emory (**G**) cohorts. **H** IF images showing the localization of survivin (green) in AA and EA TNBC cell lines. Nuclei were stained with Hoechst (blue) and tubulin (red). **I** Immunoblot showing p-survivin (S20, T117) levels in AA and EA TNBC cell lines (*n* = 3 each). FPKM fragments per kilobase of transcript per million mapped reads, TPM transcripts per million mapped reads. Bars indicate mean ± SEM. Unpaired two-tailed Student’s *t*-test with Welch’s correction was used to determine statistical significance (*P < 0.05, ***P < 0.0005, ns = non-significant). Scale bars in **E** and **H** are 100 µm and 10 µm, respectively.

Colnaghi et al. [24] and Wheatley et al. [25]. have suggested that both PLK1 and AURKB phosphorylate survivin. PLK1 phosphorylates survivin at S20, and AURKB phosphorylates survivin at T117. Survivin phosphorylation at S20 by PLK1 regulates chromosome alignment and cell proliferation, whereas phosphorylation at T117 is essential for CPC function [24, 25]. Considering these findings, we evaluated the expression levels and phosphorylation status of survivin in AA and EA TNBC cell lines and tissues. Interestingly, we did not observe any significant differences in the mRNA levels of survivin in the TCGA dataset (Fig. 2A), cell line data analyzed using Neve et al. [26] (Fig. 2B), and in-house cell lines (Fig. 2C). Similarly, we found no significant racial differences in the protein levels of survivin in TNBC cell lines (Fig. 2D), the Dekalb cohort (Fig. 2E, F), or the Emory cohort (Fig. 2G).

Survivin shuttles between the nucleus and the cytoplasm and the localization of survivin in these two subcellular compartments have been associated with different outcomes. Nuclear localization of survivin is an independent indicator of good prognosis, whereas accumulation of survivin in the cytoplasm correlates with poor prognosis in BC [27, 28]. Thus, we assessed whether survivin localization in TNBC cells differed between AAs and EAs. Surprisingly, we found no significant racial differences in the localization of survivin in the Dekalb cohort (Fig. 2F), Emory cohort (Fig. 2G), or TNBC cell lines (Fig. 2H). Survivin exhibited nuclear accumulation in both AA and EA TNBC cells.

We also investigated the phosphorylation levels of survivin at S20 and T117. Interestingly, the phosphorylation levels of survivin at S20 and T117 were higher in AA TNBC cells than in EA TNBC cells (Fig. 2I), suggesting that PTMs may contribute to racial disparities in TNBC. Furthermore, when patients were stratified based on survivin expression, overall survival was lower in AA patients with high survivin levels than in EAs with high survivin levels (Supplementary Fig. 4A–C). Moreover, survivin expression levels were positively correlated with a mitotic score, especially in AA patients with TNBC (Supplementary Fig. 4D, E). These results support the prognostic value of survivin in AA patients with TNBC.

### PLK1 and AURKB silencing or inhibition modulate p-survivin levels in AA patients with TNBC

**Fig. 3:**
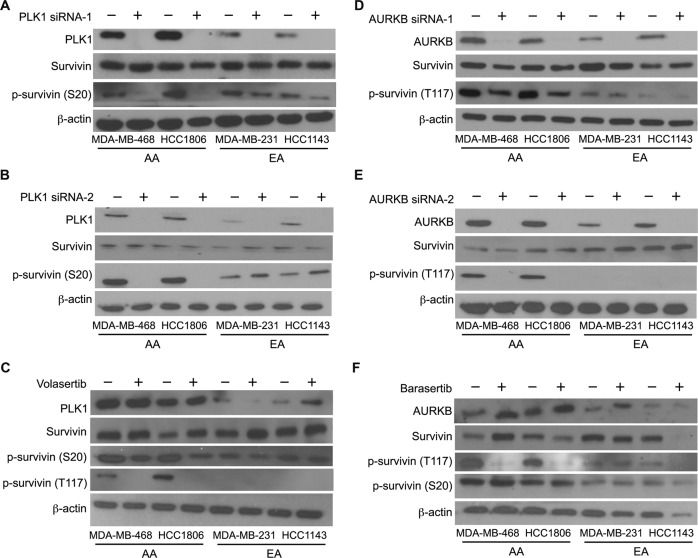
Silencing or inhibition of PLK1 and AURKB modulates survivin phosphorylation at S20 and T117 in AA TNBC cells. **A**, **B** Immunoblots showing the levels of PLK1, survivin, p-survivin (S20), and β-actin after PLK1 silencing (**A**, **B**) or inhibition (**C**) in AA and EA TNBC cell lines. **D**–**F** Immunoblots showing the levels of AURKB, survivin, p-survivin (T117), and β-actin after AURKB silencing (**D**, **E**), or inhibition (**F**) in AA and EA TNBC cell lines.

In AA TNBC cells, but not in EA TNBC cells, *PLK1* and *AURKB* KD profoundly decreased survivin phosphorylation at S20 and T117, respectively (Fig. 3A, B, D, E; Supplementary Fig. 2C–E). A similar effect was observed in TNBC cells treated with the PLK1 and AURKB inhibitors volasertib and barasertib, respectively (Fig. 3C, F; Supplementary Fig. 2G, H). Volasertib inhibition affected the phosphorylation of survivin at S20 and T117, whereas barasertib inhibited phosphorylation only at T117. These results indicate that AA TNBC cells rely on PLK1 and AURKB for survivin phosphorylation and that phosphorylation of survivin at S20 may influence phosphorylation at T117 in AA TNBC cells. Furthermore, PLK1 and AURKB OE in AA and EA TNBC cells (*n* = 3 each) resulted in significant upregulation of S20 or T117, particularly in EA TNBCs, which have a minimum basal level of phosphorylation at S20 and T117. The basal level of survivin remained unaltered upon PLK1 or AURKB OE (Supplementary Fig. 3A–D).

### Survivin is crucial for cell proliferation and cell cycle progression in AA TNBC cells

**Fig. 4:**
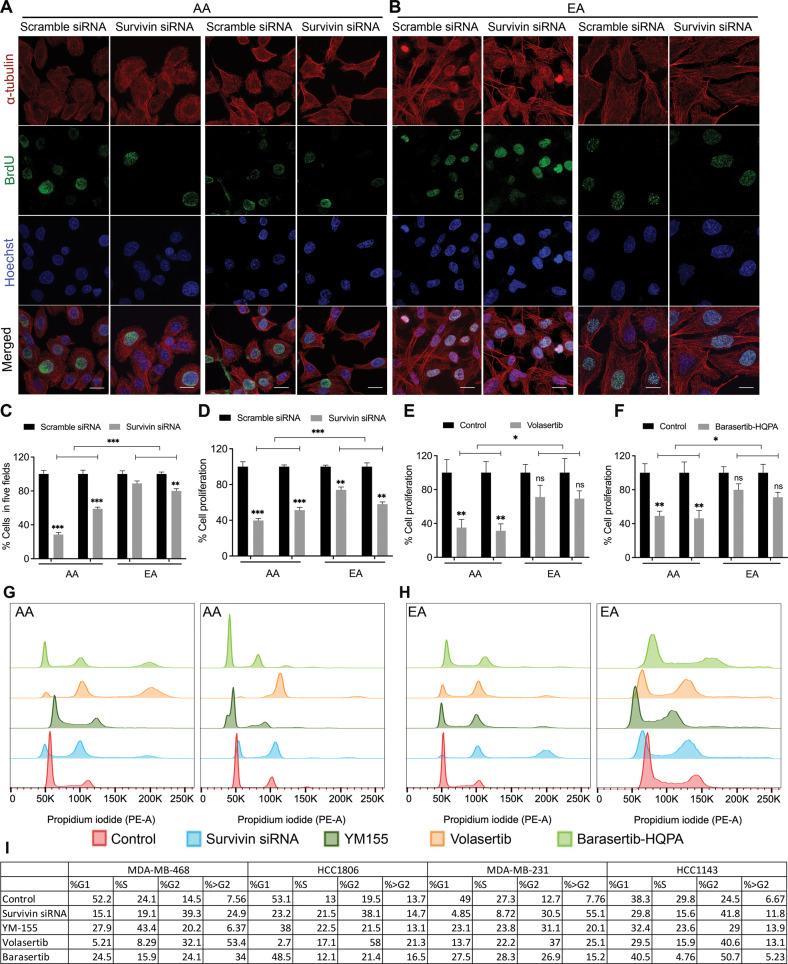
Survivin is crucial for cell proliferation and cell cycle progression in AA TNBC cells. **A**–**C** Representative immunofluorescence images (**A**, **B**) and quantification bar graphs (**C**) showing BrdU (green) incorporation in various AA (**A**) and EA (**B**) TNBC cell lines transfected with scrambled or survivin siRNAs. Nuclei were counterstained with Hoechst (blue) and tubulin (red). **D**–**F** Bar graphs showing BrdU incorporation in AA and EA TNBC cells treated with survivin siRNA (**D**), volasertib (**E**), and barasertib-HQPA (**F**). Absorbance was measured at 450–540 nm. **G**–**I** Flow cytometry analysis depicting various cell cycle phases in AA (**G**) and EA (**H**) TNBC cells treated with control (red), survivin siRNA (light blue), YM155 (dark green), volasertib (orange), and barasertib (bright green) and their quantification (**I**). Data were analyzed using FlowoJo. Bars indicate mean ± SEM. Unpaired two-tailed Student’s *t*-test with Welch’s correction was used to determine statistical significance (*P < 0.05, ***P < 0.0005, ns = non-significant). The scale bar represents 10 µm.

Next, we evaluated the functional activity of survivin in AA and EA TNBC cell lines. To this end, we transfected AA and EA TNBC cell lines with siRNAs specific for survivin to silence its expression. KD efficiency in all transfected TNBC cell lines was 95–100% (Supplementary Fig. 5A). BrdU cell proliferation assay revealed that BrdU incorporation was significantly decreased upon survivin KD in AA but not in EA TNBC cells (Fig. 4A–D; Supplementary Fig. 5F). These results indicate that, despite the similar expression level of survivin in AA and EA TNBC cells, the functional activity of survivin differs considerably between EAs and AAs, with AA TNBC cells being more dependent on survivin for cell proliferation than EA TNBC cells. Consistently, treatment with volasertib and barasertib-HQPA significantly reduced TNBC cell proliferation in AA cells but not in EA cells (Fig. 4E, F; Supplementary Fig. 5G, H). Survivin is crucial for cell cycle progression, as it regulates progression to the G2/M phase of the cell cycle [29, 30]. We performed cell cycle analysis to identify racial differences in the importance of survivin in TNBC cell kinetics. We found that AA TNBC cells were more proliferative than EA TNBC cells (Supplementary Fig. 4B–E). Survivin KD significantly decreased the percentage of cells in the G0/G1 phase and increased the percentage of cells in the G2/M phase in AA TNBC cells; changes in cycle kinetics in EA TNBC cells were minimal (Fig. 4G–I; Supplementary Fig. 6A–D).

We also performed cell cycle analysis after treatment with YM155 (survivin inhibitor), volasertib, and barasertib. We observed a higher percentage of cells in the G2/M phase under all conditions, particularly in AA TNBC cells (Fig. 4G–I). The higher percentage of AA TNBC cells arrested in the G2/M phase suggests that these cells cannot complete mitosis or proliferate. The modulation of the cell cycle in AA TNBC cells upon treatment with volasertib and barasertib may stem from the lower levels of survivin phosphorylation at S20 and T117 due to the inhibition of PLK1 and AURKB. We also investigated the role of survivin in cell invasion and migration in AA and EA TNBC cells. Survivin silencing did not significantly affect cell invasion or migration in any of the cell lines (Supplementary Fig. 7A–D). Taken together, these data suggest that survivin, PLK1, and AURKB are crucial for cell proliferation and cell cycle progression in AA but not in EA TNBC cells and may serve as viable therapeutic targets in AA patients with TNBC.

### Inhibition of PLK1 and AURKB suppresses tumor growth and prolongs survival in mice bearing AA TNBC tumors

**Fig. 5:**
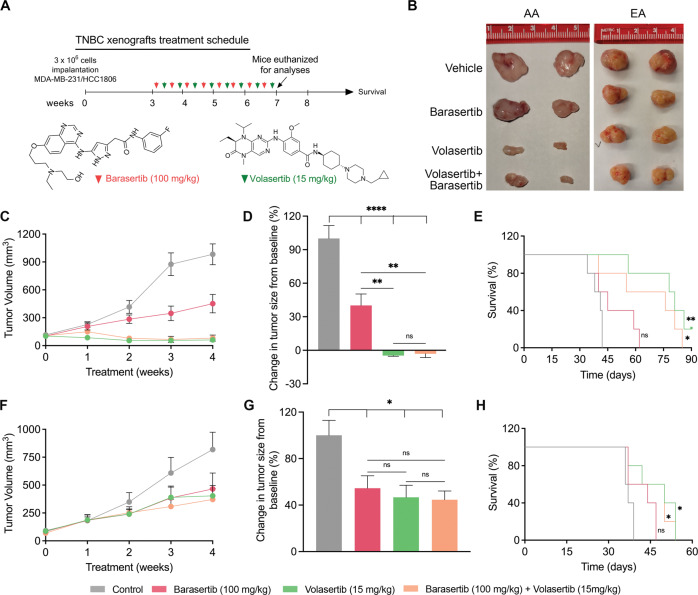
Inhibition of PLK1 and AURKB suppresses tumor growth and improves survival in nude mice bearing AA TNBC tumors. **A** Schematic diagram showing the treatment schedule for volasertib (green arrow) and barasertib (red arrow) in mice bearing AA and EA TNBC xenografts. **B**–**F** Representative tumor images (**B**), changes in tumor volume in AA (**C**) and EA (**F**) tumors, and tumor growth inhibition in mice with AA (*n* = 12) (**D**) and EA (*n* = 12) (**G**) TNBC xenografts. **E**, **H** Kaplan–Meier plots showing survival in mice bearing AA (*n* = 12) (**E**) and EA (*n* = 12) (**H**) xenografts. Bars indicate mean ± SEM. Unpaired two-tailed Student’s *t*-test with Welch’s correction was used to determine statistical significance (*P < 0.05, **P < 0.005, ****P < 0.00005, ns = non-significant).

To validate our in vitro findings in a preclinical mouse model, we divided mice bearing AA and EA TNBC tumors into four treatment groups: vehicle, volasertib, barasertib, and volasertib plus barasertib. All mice were monitored for four weeks after the start of the treatment. After treatment, a few mice from each group were kept for survival analysis, and the remaining mice were euthanized for tumor collection (Fig. 5A). We did not observe any differences in the body weight of mice in any of the treatment groups (Supplementary Fig. 8A, B), suggesting an excellent safety profile for volasertib and barasertib. Interestingly, treatment with volasertib, barasertib, and their combination significantly decreased tumor volume and size in mice bearing AA TNBC xenografts (Fig. 5B–D) but not in those with EA TNBC tumors (Fig. 5B, F, G). These data further support that AA TNBCs are more reliant on PLK1 and AURKB for cell proliferation and that their inhibition using volasertib and barasertib can attenuate the growth of these tumors. Survival analysis showed a significant prolongation in the survival of mice bearing AA TNBC xenografts treated with volasertib (up to 90 days), barasertib (up to 60 days), or their combination (up to 90 days; Fig. 5E). Although the survival of mice bearing EA TNBC xenografts was prolonged by all treatments (Fig. 5H), the survival benefit of these mice was less profound than that of mice bearing AA TNBC xenografts. Collectively, these results provide compelling evidence that volasertib and barasertib may improve the disease course in AA patients with TNBC.

### Inhibition of PLK1 and AURKB decreases Ki-67 and p-survivin levels in mice bearing AA TNBC tumors

**Fig. 6:**
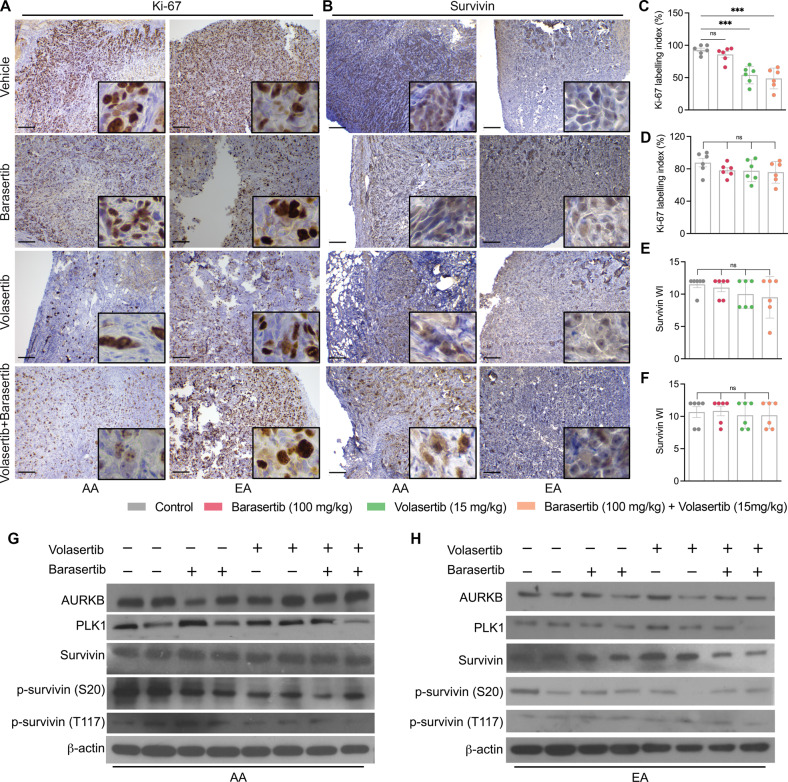
Inhibition of PLK1 and AURKB decreases Ki-67 and p-survivin levels in mice bearing AA TNBC tumors. **A**–**F** Representative IHC images (**A**, **B**) and bar graphs (**C**–**F**) showing Ki-67 and survivin levels in AA (**C**, **E**) and EA (**D**, **F**) TNBC xenografts under various treatment conditions. **G**, **H** Immunoblots showing the levels of p-survivin (T117), p-survivin (S20), total survivin, AURKB, PLK1, and β-actin in AA (**G**) and EA (**H**) fresh-frozen xenograft tumor lysates from mice treated with volasertib, barasertib, or their combination (*n* = 12 per treatment group). Bars represent mean ± SEM. Unpaired two-tailed Student’s *t*-test with Welch’s correction was used to determine statistical significance (****P < 0.00005, ns = non-significant). The scale bar represents 100 µm.

We assessed the effects of volasertib and barasertib on the levels of Ki-67 and p-survivin (S20 and T117) in TNBC xenografts and observed a significant decrease in Ki-67 levels in AA TNBC xenografts when mice were treated with volasertib, barasertib, or their combination (Fig. 6A, C). However, Ki-67 levels remained unaltered in EA TNBC xenografts (Fig. 6A, D). As expected, there were no differences in total survivin levels (Fig. 6B, E–H), PLK1 (Fig. 6G, H; Suppl. Fig. 8C, E, F), and AURKB (Fig. 6G, H; Supplementary Fig. 8D, G, H) in AA or EA TNBC xenografts. However, p-survivin (S20 and T117) levels were significantly decreased upon volasertib and barasertib treatment, especially in mice bearing AA TNBC tumors (Fig. 6G, H). These results are in line with our in vitro data and suggest that higher phosphorylation levels of survivin are linked to increased tumor progression in AA patients with TNBC. These findings also support the idea that targeting PLK1 and AURKB using volasertib and barasertib represents a promising therapeutic strategy for AA patients with TNBC.

### CPC complex formation is highest in S20-T117 double phospho-mimic mutants

**Fig. 7:**
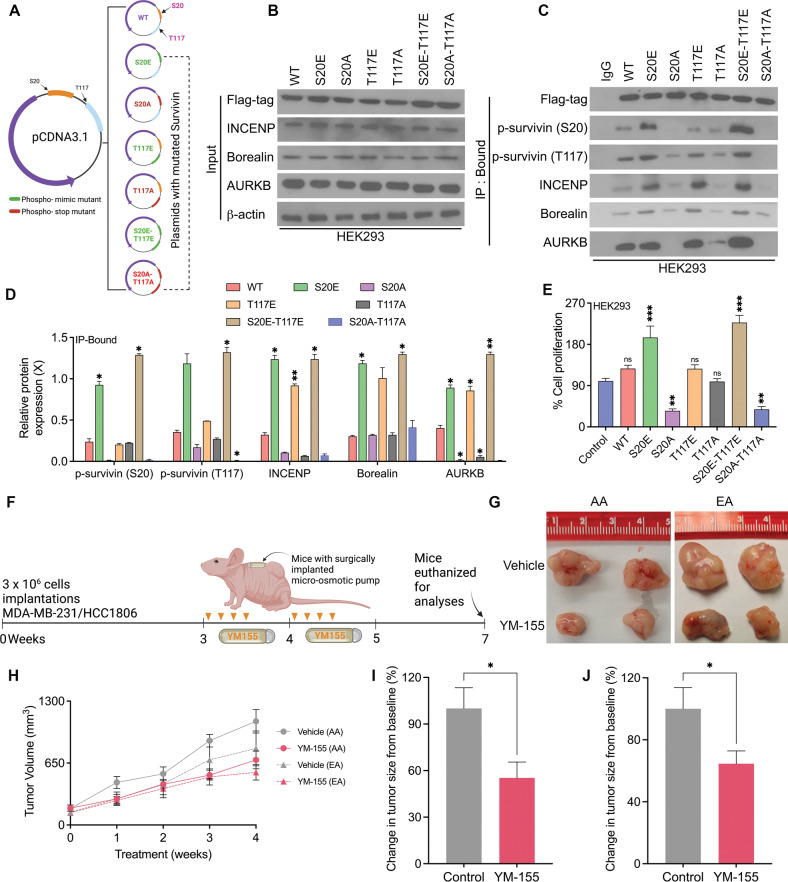
CPC complex formation is highest in S20-T117 double phospho-mimic survivin mutants. **A** Schematic representation of survivin-mutant plasmids. **B**–**D** Immunoblots (**B**, **C**) showing the levels of CPC proteins in input (**B**) and IP-bound (**C**) samples from cells expressing various survivin-WT and mutant plasmids, and their respective quantification (**D**). **E** Bar graphs showing the percentage of cell proliferation in control cells and in cells expressing survivin-WT and mutant plasmids. **F** Schematic illustration of YM155 treatment schedule in mice bearing tumors and surgically implanted with osmotic pumps. **G**–**J** Representative tumor images (**G**), changes in tumor volume (**H**), and changes in tumor size (**I**, **J**) in mice bearing AA (*n* = 12) and EA (*n* = 12) TNBC xenografts. Bars represent mean ± SEM. Unpaired two-tailed Student’s *t*-test with Welch’s correction was used to determine statistical significance (*P < 0.05, **P < 0.005, ns = non-significant).

Because PLK1 silencing or inhibition caused a significant decrease in the phosphorylation levels of survivin at S20 and T117, we speculated that S20 phosphorylation drives T117 phosphorylation by AURKB. To test this hypothesis, we designed plasmids for WT survivin and phospho-stop (A-alanine) mutants (S20, T117, and their combination). We also generated phospho-mimic (E-glutamine) mutants for S20 and T117 (Fig. 7A). Six different survivin phosphor-variants were analyzed (S20E, S20A, T117E, T117A, S20E-T117E, S20A-T117A) in HEK293 cells with stable OE of PLK1 and AURKB (Supplementary Fig. 9A). IP analysis revealed that interaction of CPC proteins (including survivin, AURKB, Borealin, and INCENP) was significantly higher in survivin with single and double phospho-mimic mutants (S20E, T117E, S20E-T117E) than in single and double phospho-stop mutants (S20A, T117A, S20A-T117A; Fig. 7C, D). Input samples that did not go through IP were used for comparison (Fig. 7B). The highest binding of CPC proteins was observed in the double phospho-mimic mutants (S20E-T117E), implying that phosphorylation of both S20 and T117 is necessary for CPC complex formation, resulting in early entry into anaphase. We also assessed the effects of survivin phosphorylation at S20 and T117 on cell proliferation. Cells expressing the double phospho-mimic mutants exhibited the highest percentage of cell proliferation (Fig. 7E), followed by cells expressing the single phospho-mimic S20 mutant. Our phospho-mimic variant analyses suggest that phosphorylation of survivin at S20 by PLK1 drives the binding of AURKB to survivin, leading to phosphorylation at T117. Phosphorylation of survivin at both S20 and T117 regulates CPC formation, thereby promoting cell proliferation.

Next, we assessed whether treatment with the survivin inhibitor YM155 could decrease the growth of AA TNBC tumors. Upon YM155 treatment, no significant difference in body weight was observed in either group (Supplementary Fig. 9B, C). YM155 treatment caused a more profound decrease in the volume and size of AA TNBC tumors than of EA tumors (Fig. 7G–J). However, the ability of YM155 to suppress the growth of TNBC xenografts was weaker than that of volasertib and barasertib. These data strongly suggest that cancer cells with survivin inhibition at the transcriptional level due to YM155 treatment may have compensatory mechanisms to overcome the loss of survivin expression by enhancing the activity of the remaining survivin.

## Discussion

Tumor heterogeneity and the lack of predictive biomarkers and therapeutic targets in TNBC contribute to the poor survival of patients with TNBC. As TNBC lacks the expression of hormone receptors, patients with TNBC have limited treatment options. The disparate racial burden is evident within the TNBC subtype, with AA women exhibiting a much more aggressive disease course, poorer prognosis, and lower survival rates than women of European descent [2, 3]. The disparate burden of the disease accentuates the critical need to identify novel drug targets and biomarkers to risk-stratify patients and predict prognosis.

This study provides strong evidence that TNBC disparities go beyond differences in gene expression and protein levels and that differential PTM profiles play a crucial role in racial inequality in TNBC. A recent study by Golavilli et al. showed that in TNBC, AMP-activated protein kinase (AMPK) activated glycogen synthase kinase 3 beta (GSK3β) and Sirtuin 1 (SIRT1) by inhibiting their phosphorylation at Ser9 and Ser47, respectively. In turn, the activation of GSK3β and SIRT1 downregulated metadherin (MTDH) and inhibited TNBC cell proliferation [31]. Hanigan et al. demonstrated that c-Jun N-terminal kinase (JNK)-mediated histone deacetylase 3 (HDAC3) phosphorylation in TNBC cells was essential for HDAC inhibitor binding and selectivity [32]. Moreover, Hsu et al. revealed that glycosylation, phosphorylation, ubiquitination, SUMOylation, and acetylation played essential roles in the regulation of PD-L1 stability, translocation, and interaction with other proteins. Aberrant PTMs have also been implicated in PD-L1-mediated immune resistance in TNBC [33]. Collectively, these studies suggest a crucial role for PTMs in TNBC development and progression. Thus, modulating PTMs using small molecule inhibitors and monoclonal antibodies has emerged as an attractive anticancer approach [34, 35]. However, to the best of our knowledge, no studies have shown the role of PTMs in racial disparities in TNBC.

Our in silico analyses revealed significantly higher expression levels of PLK1 and AURKB in AA patients with TNBC than in their EA counterparts. Survivin, an IAP family member and substrate of PLK1 and AURKB, is involved in carcinogenesis, tumor progression, cancer cell proliferation, inhibition of apoptosis, neoangiogenesis, and drug resistance [36, 37]. Because of the higher expression of survivin in cancer cells than in normal tissues, modulating the expression and function of survivin in cancer cells may have little to no toxic effects on the surrounding normal tissues. Zhang et al. [16] reported a significant correlation between survivin levels and tumor size, lymph node metastasis, and poor survival in patients with TNBC. However, the differential function of survivin in racially diverse TNBC populations has remained unappreciated.

Despite the similar expression levels and localization patterns of survivin in AA and EA patients with TNBC, survivin silencing or inhibition inhibited cell proliferation and cell cycle progression exclusively in AA TNBC cells. Our data suggest that survivin phosphorylation at S20 and T117 by PLK1 and AURKB is essential for tumor progression in AA patients with TNBC and contributes to racial disparities in TNBC. This is the first study to propose that in AAs, TNBCs rely on survivin phosphorylation by PLK1 and AURKB for cell proliferation and cell cycle progression. PLK1 and AURKB inhibition using small molecule inhibitors inhibited survivin phosphorylation at S20 and T117, respectively, reducing proliferation in AA TNBC cells. Additionally, AA TNBC cell lines showed higher levels of p-survivin S20 and p-survivin T117 than EA TNBC cells. The higher phosphorylation of survivin at S20 and T117 in AA TNBC cells is responsible for higher CPC complex formation and, consequently, a higher proliferation rate. Our data also suggest that in contrast to the inhibition of survivin phosphorylation using volasertib and barasertib, inhibition of survivin at the transcriptional level using YM155 is not sufficient to suppress tumor growth in AAs with TNBC. Thus, targeting survivin phosphorylation at S20 and T117 using volasertib and barasertib may serve as a viable treatment alternative for AA patients with TNBC. Studies to further explore the role of p-survivin in BC-related racial disparities are currently underway in our laboratory. Additionally, in-depth analyses are warranted to provide further insights into the role of survivin phosphorylation in changes to its scaffolding ability, which affects CPC formation.

This study has some limitations. There is a lack of in-house datasets to evaluate the expression levels of various kinases in AA and EA patients with TNBC; hence, data from one publicly available dataset were used in this study. Validation of our findings in additional datasets is required. Due to the limited number of available TNBC cell models from AA patients and their inability to form tumors in xenograft mouse models, only one AA and one EA TNBC cell line were used for the in vivo study. Although our in vivo data are compelling, future in vivo studies using additional AA and EA TNBC cell lines with similar drug responses or growth kinetics are necessary.

Overall, to our knowledge, this is the first study to demonstrate the role of survivin phosphorylation in dictating disparate tumor outcomes within a racially divergent TNBC subpopulation. Our in vitro and in vivo findings suggest that the phosphorylation of survivin by PLK1 and AURKB promotes tumor cell proliferation and cell cycle progression in AA TNBC cells but not in EA TNBC cells. Future investigations into the role of PTMs in racial disparities in BC may guide the development of new therapies for TNBC.

## Supplementary information

Supplementary figures 1-9

All raw WB old and revised

Checklist

## Data Availability

The data of this study will be shared by the corresponding author (R.A.) upon reasonable request.
