# Supplementary material for: PLK1 and AURKB phosphorylate survivin differentially to affect proliferation in racially distinct triple-negative breast cancer
Source: Cell Death Dis. 2023 Jan 10;14(1):12. doi: 10.1038/s41419-022-05539-5 (PMC9832024; doi:10.1038/s41419-022-05539-5)
Supplement: Supplementary file 1 — Supplementary figures 1-9 [file 41419_2022_5539_MOESM1_ESM.pdf]

Supplementary figures

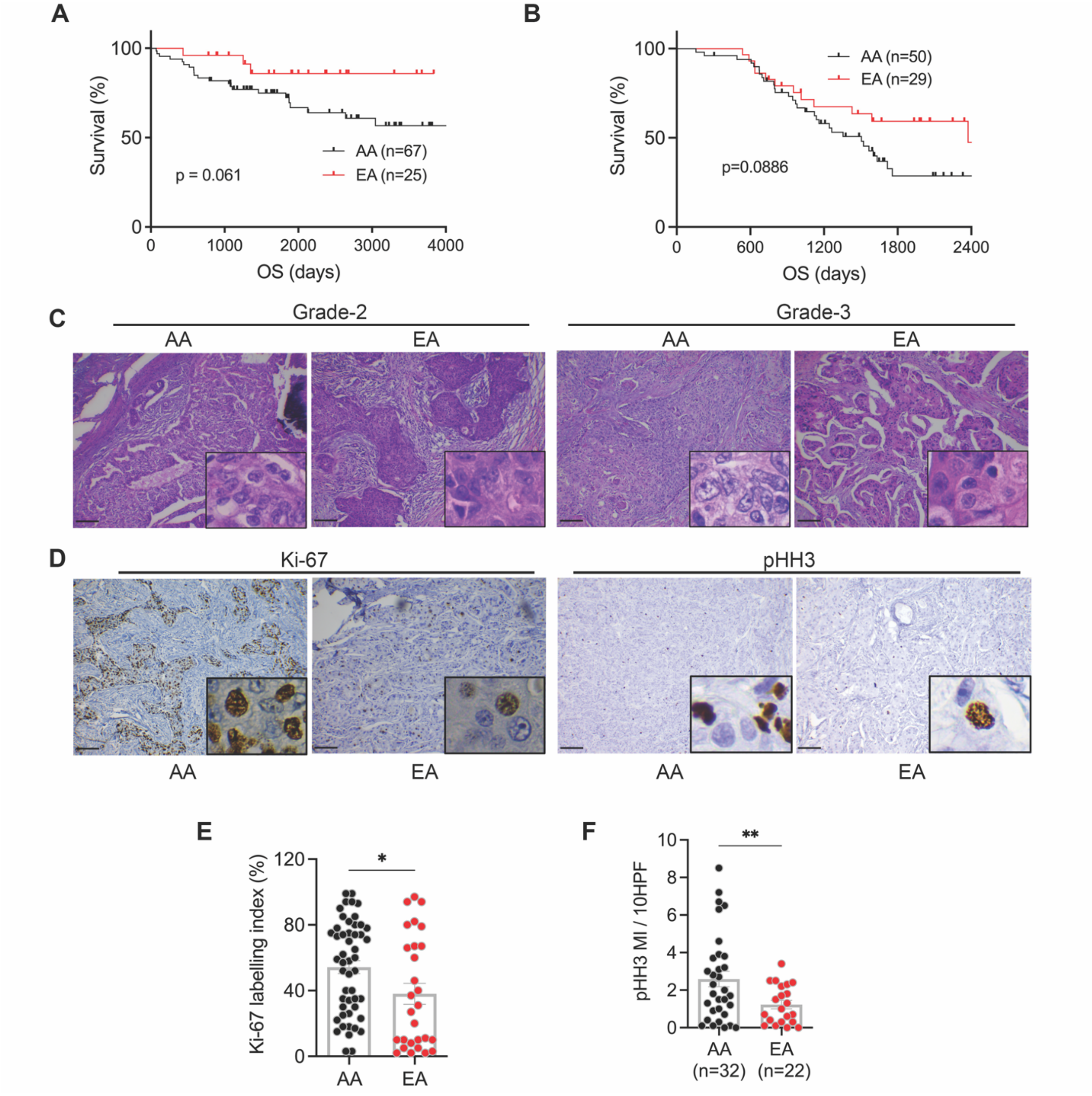

Supplementary Figure 1. AA patients with TNBC have lower survival rates and higher proliferation index than EAs.

(A–B) Kaplan-Meier plot showing the relationship between ancestry (self-reported) and survival in AA and EA TNBC patients from Emory (A) and Dekalb (B) hospitals. (C) Representative H&E images showing grade 2 and 3 TNBC tissues from AA and EA patients. (D, G, H) Representative

IHC images (D) and quantification of Ki-67 (G) and pHH3 (H) in grade- and stage-matched AA and EA patients with TNBC from Dekalb hospital. (E, F) Bar graphs showing mitosis scores in the Emory (E) and Dekalb (F) cohorts. Bars indicate mean ± SEM. Log-rank (Mantel-Cox) and unpaired two-tailed Student's *t*-test with Welch's correction was used to determine statistical significance (**P*<0.05, *****P*< 0.00005, ns=non-significant). The scale bar represents 100 µm.

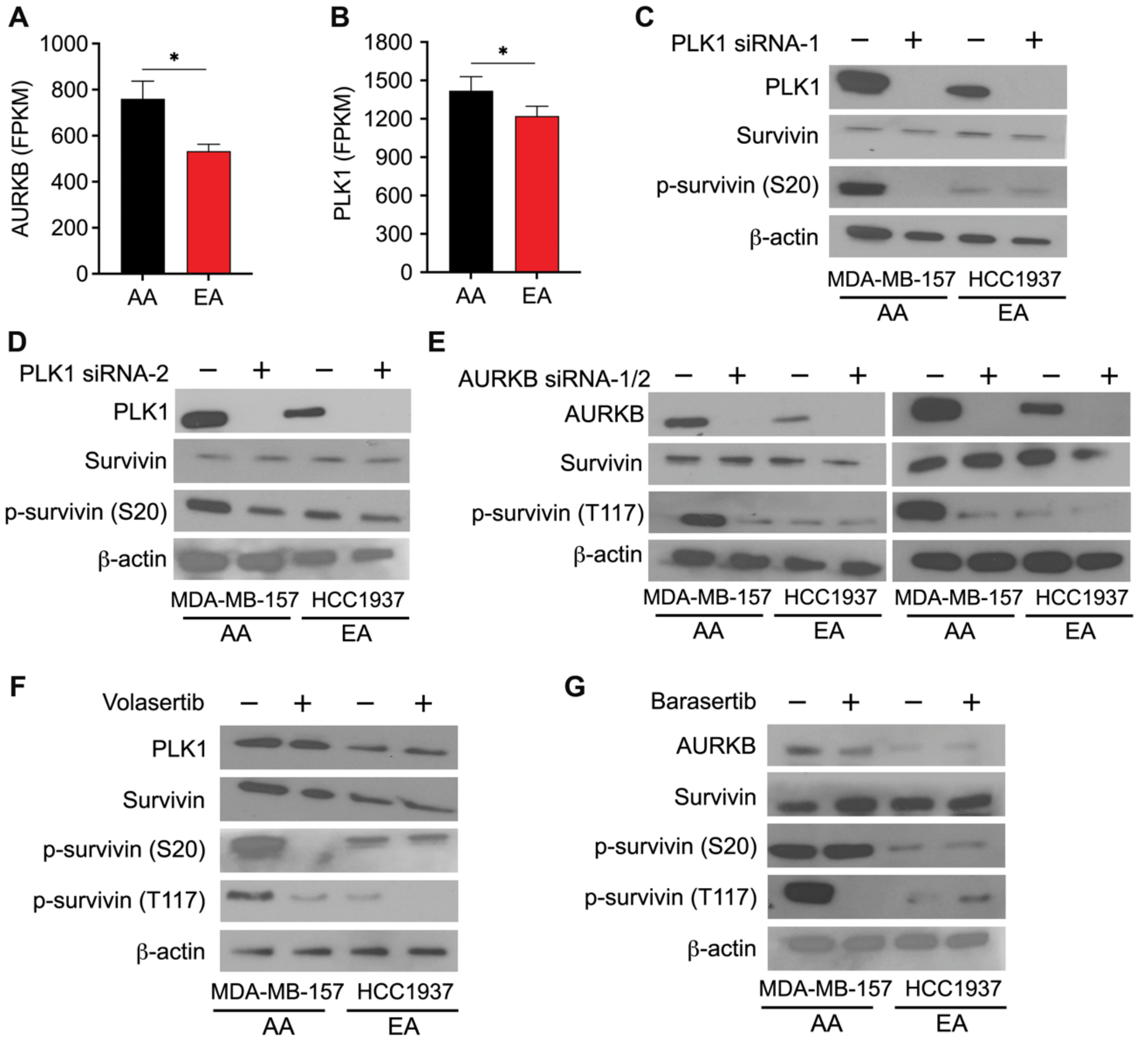

Supplementary Figure 2. Effects of PLK1 and AURKB ablation on survivin phosphorylation. (A, B) Bar graphs showing *AURKB* (A) and *PLK1* (B) expression levels in AA and EA patients with TNBC (TCGA dataset; filtered for TNBC). Immunoblots showing the levels

of survivin (total, S20, and T117) upon PLK1 silencing (C, D) and inhibition (E) in AA (n=1) and EA (n=1) TNBC cells. Immunoblots showing the levels of total survivin and p-survivin (S20 and T117) after AURKB silencing (F, G) and inhibition (H) in AA (n=1) and EA (n=1) TNBC cells.

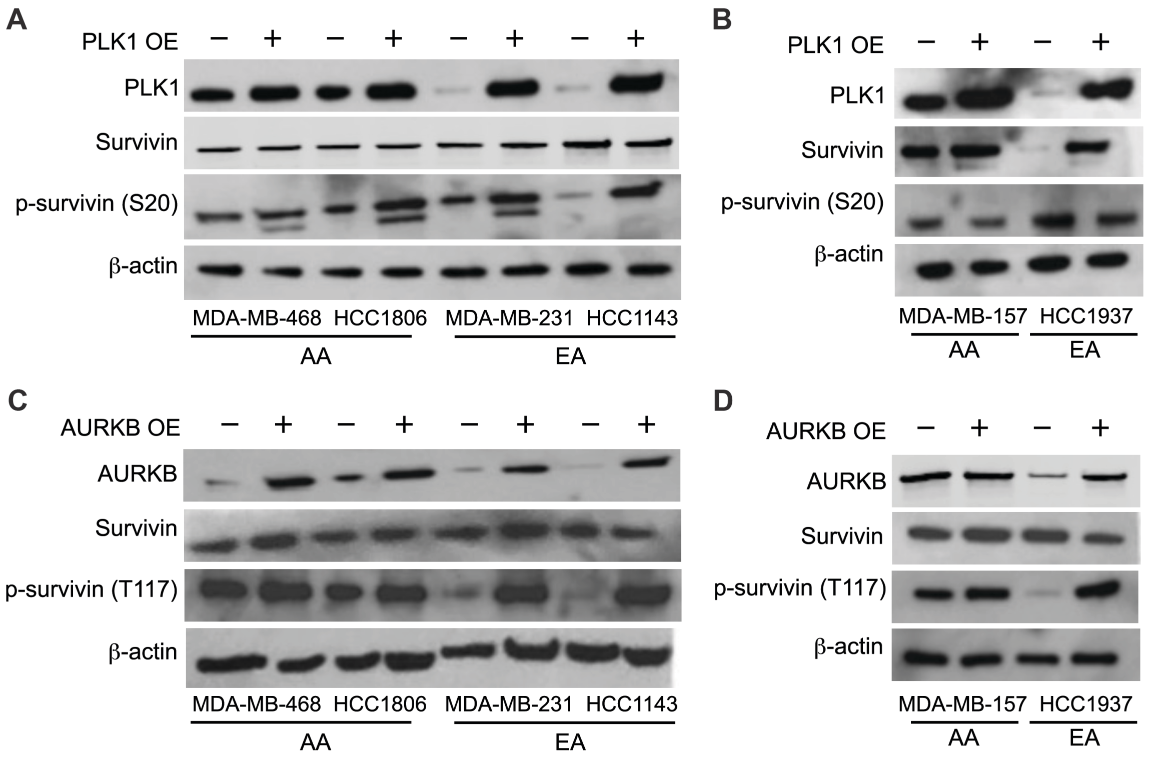

Supplementary Figure 3. Effects of PLK1 and AURKB overexpression on survivin phosphorylation. (A–D) Immunoblots showing the levels of total survivin and p-survivin (S20, T117) upon PLK1 (A, B) and AURKB (C, D) overexpression in AA (n=3) and EA (n=3) TNBC cells.

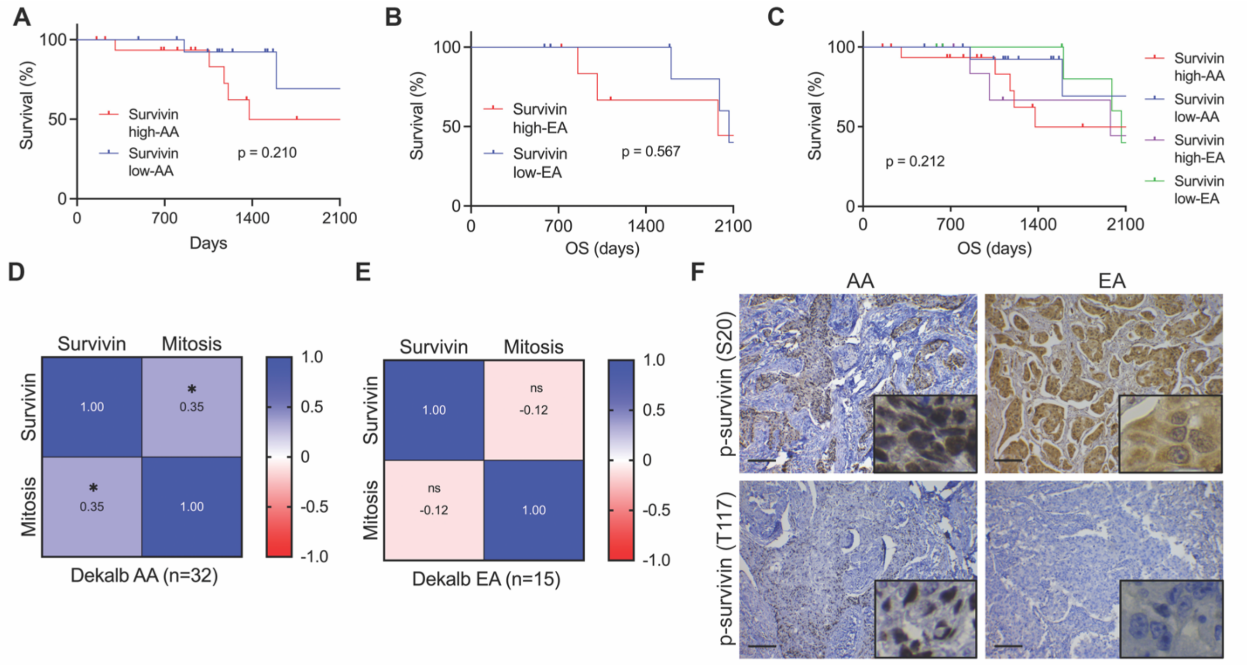

Supplementary Figure 4. Survivin levels predict prognosis in AA patients with TNBC and are correlated with mitosis scores in EA and AA patients.

(A–C) Kaplan-Meier plots showing the relationship between survivin levels and survival in AA (n=32) (A), EA (n=16) (B), and all (C) patients. (D, E) Positive correlation between survivin levels and mitotic score in AA (D) and EA (E) TNBC patients (Dekalb cohort). (F) Representative IHC images of p-survivin (S20 and T117) in the Dekalb cohort. Bars indicate mean ± SEM. Unpaired two-tailed Student's *t*-test with Welch's correction was used to determine statistical significance (*P<0.05, ns=non-significant). The scale bar represents 100 µm.

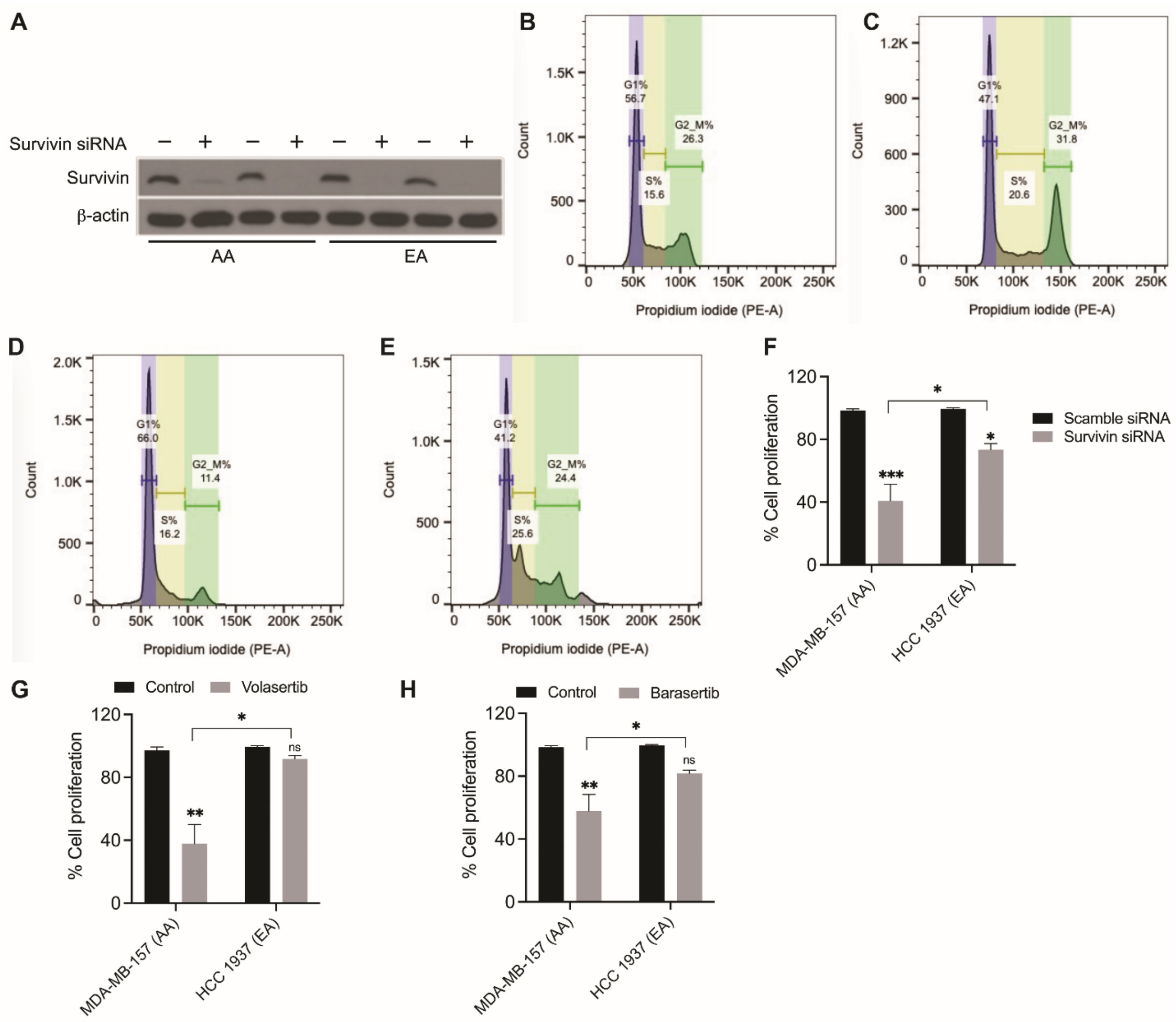

Supplementary Figure 5. Survivin KD efficiency and cell cycle kinetics in TNBC cell lines. (A) Immunoblot showing survivin KD efficiency in AA and EA TNBC cell lines. (B–E) Flow cytometry analysis depicting cell cycle kinetics in MFM223 (B), HCC70 (C), BT-549 (D), and BT-20 (E) TNBC cells. Cell cycle analysis was performed using FlowJo. (F–H) Bar graphs showing cell proliferation assessed using the BrdU incorporation assay in TNBC cells treated with survivin siRNAs (F), volasertib (G), and barasertib (H). Absorbance was measured at 450–540 nm. Bars indicate mean ± SEM. Unpaired two-tailed Student's *t*-test with Welch's correction was used to determine statistical significance (*P<0.05, ***P<0.0005, ns=non-significant).

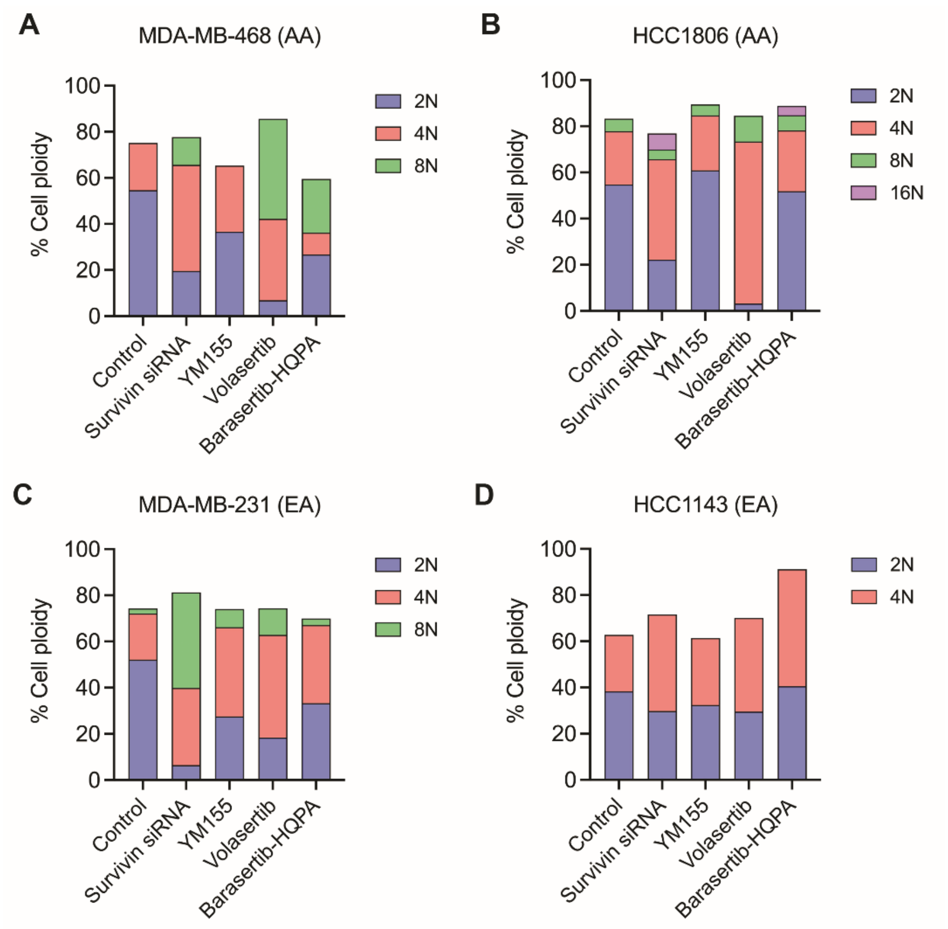

Supplementary Figure 6. Cell cycle analysis in AA and EA TNBC cell lines.

(A–D) Bar graphs showing the percentages of cells in each cell cycle phases (presented in Fig. 4G, H) showing various ploidies in AA (A, B) and EA (C, D) cell lines.

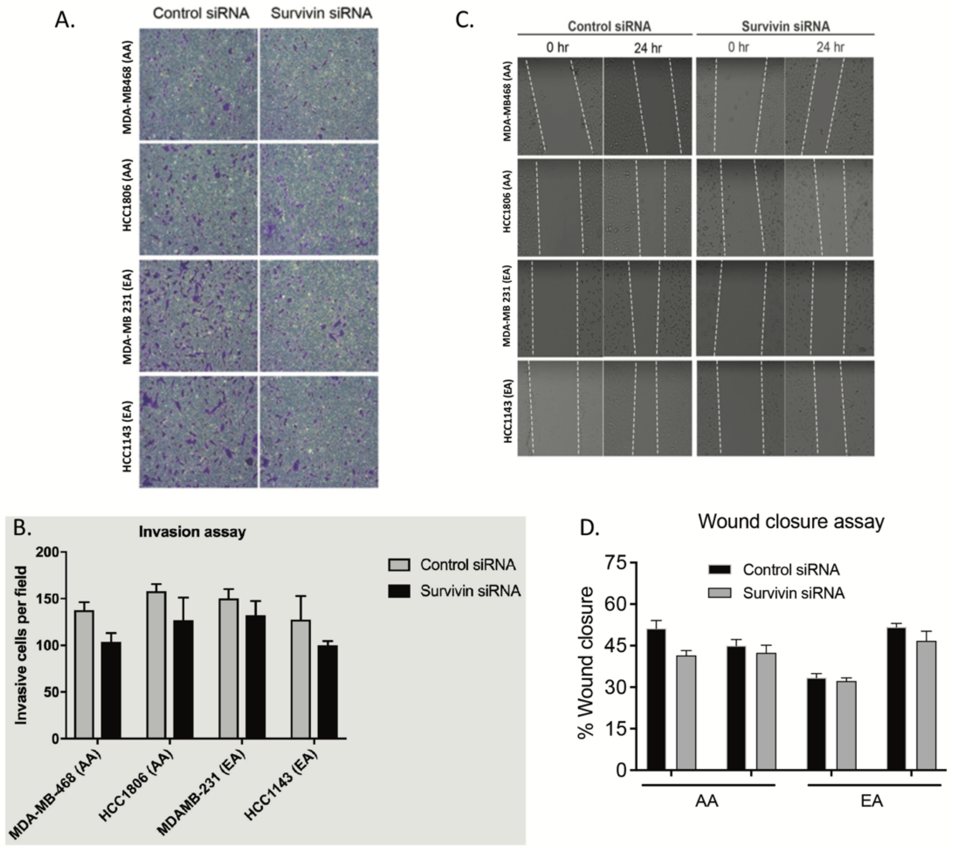

Supplementary Figure 7. Survivin silencing does not impair cell invasion and migration in TNBC cell lines.

(A, B) Representative images (A) and quantification (B) of cell invasion (purple) in AA and EA TNBC cell lines transfected with control or survivin siRNAs. Data were analyzed using ImageJ. (C, D) Representative wound closure images (C) and quantification plots (D) showing the percentage of wound closure in AA and EA TNBC cell lines transfected with control or survivin siRNAs. Data were analyzed using Adobe Photoshop. Bars indicate mean ± SEM. Unpaired two-tailed Student's *t*-test with Welch's correction was used to determine statistical significance (ns=non-significant).

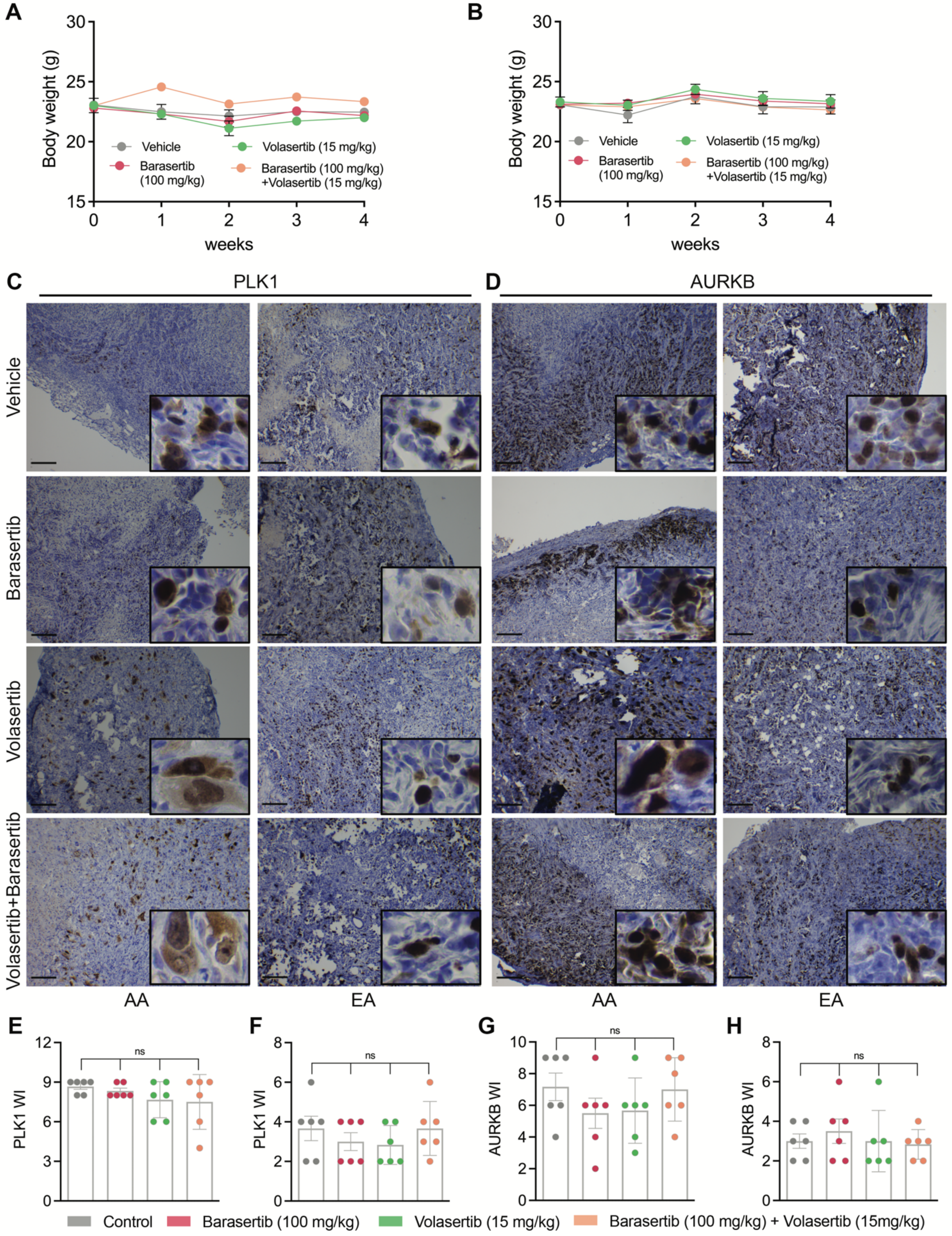
A
B
Body weight (g)
weeks
Vehicle
Barasertib (100 mg/kg)
Volasertib (15 mg/kg)
Barasertib (100 mg/kg) +Volasertib (15 mg/kg)
C
PLK1
D
AURKB
Vehicle
Barasertib
Volasertib
Volasertib+Barasertib
AA
EA
AA
EA
E
F
G
H
PLK1 WI
AURKB WI
ns
Control
Barasertib (100 mg/kg)
Volasertib (15 mg/kg)
Barasertib (100 mg/kg) + Volasertib (15mg/kg)

Supplementary Figure 8. Expression levels of PLK1 and AURKB in AA and EA TNBC xenografts under various treatment conditions.

(A, B) Graphs showing bodyweights of mice bearing AA (A) and EA (B) TNBC xenografts under different treatment conditions. (C–F) Representative IHC images of PLK1 (C) and AURKB (D) and their respective quantification in AA (E, G) and EA (F, H) TNBC xenografts (n=12 per treatment group). Bars indicate mean ± SEM. Unpaired two-tailed Student's *t*-test with Welch's correction was used to determine statistical significance (ns=non-significant). The scale bar represents 100 µm.

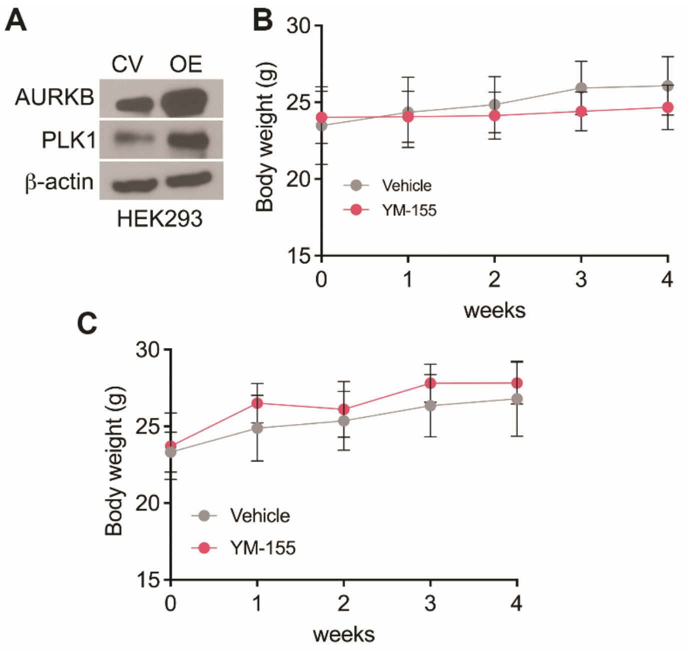

Supplementary Figure 9. YM155 treatment does not affect body weight in mice bearing TNBC tumors.

(A) Immunoblot showing stable overexpression of AURKB and PLK1 in HEK293 cells. (B, C) Graphs showing changes in body weight in mice bearing AA (B) and EA (C) TNBC tumors treated with vehicle or YM155 (n=12 per group). Bars indicate mean ± SEM. Unpaired two-tailed

Student's *t*-test with Welch's correction was used to determine statistical significance (ns=non-significant).
