# Supplementary material for: PLK1 and AURKB phosphorylate survivin differentially to affect proliferation in racially distinct triple-negative breast cancer
Source: Cell Death Dis. 2023 Jan 10;14(1):12. doi: 10.1038/s41419-022-05539-5 (PMC9832024; doi:10.1038/s41419-022-05539-5)

Figure-1I: AURKB

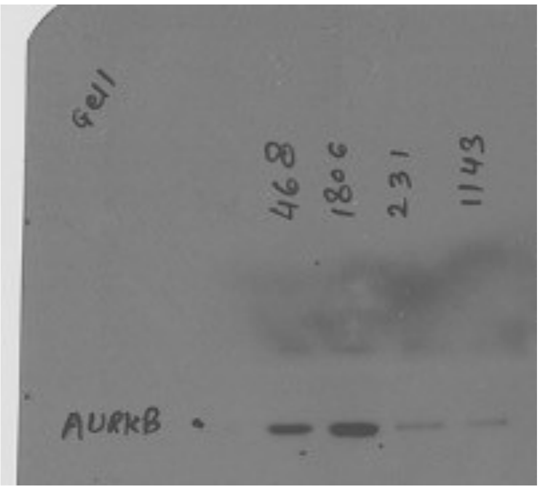

Figure-1I: Beta-actin

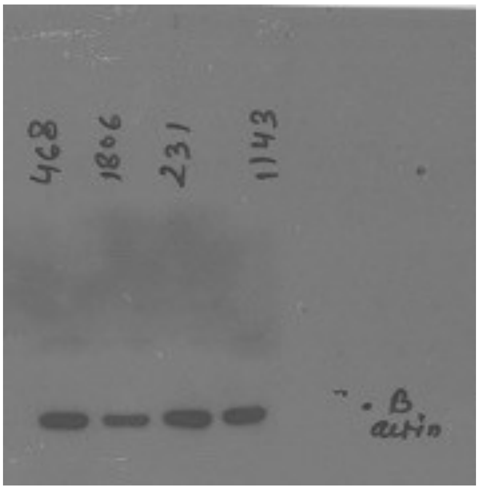

Figure-1I: PLK1

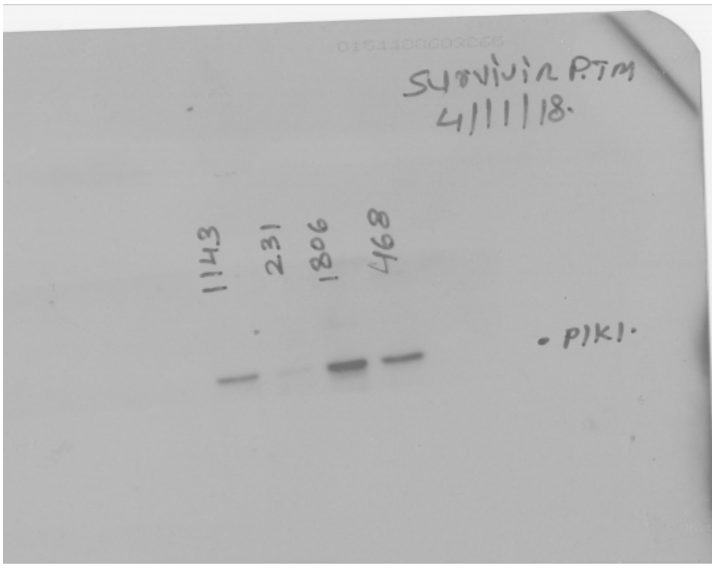

Figure-2D: Beta-actin and Survivin

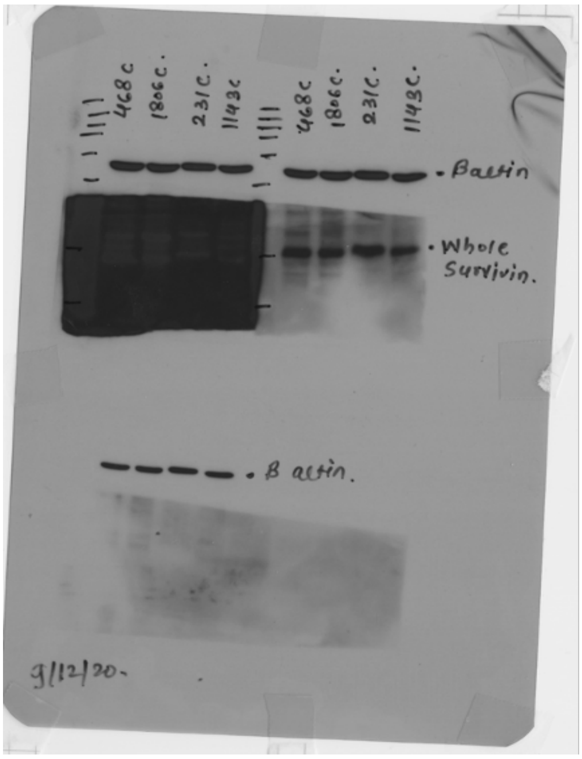

Figure-2I: Beta-actin and Survivin S-20

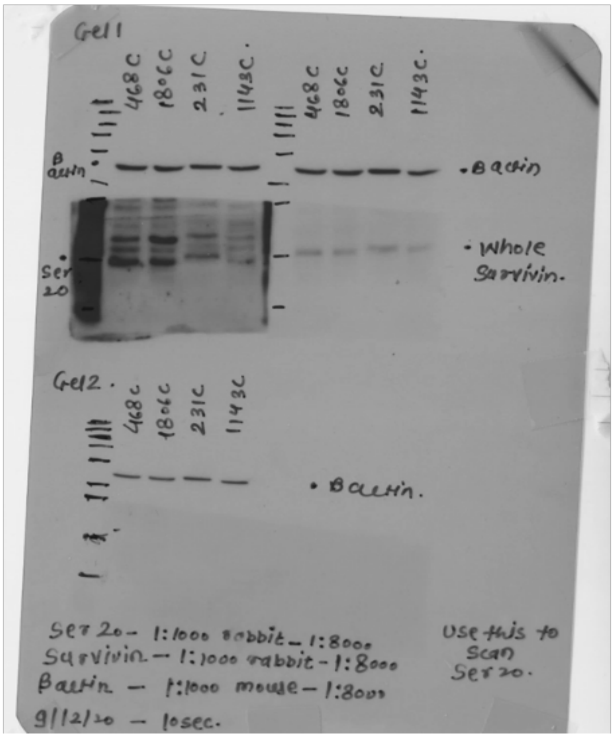

Figure-2I: Survivin T-117

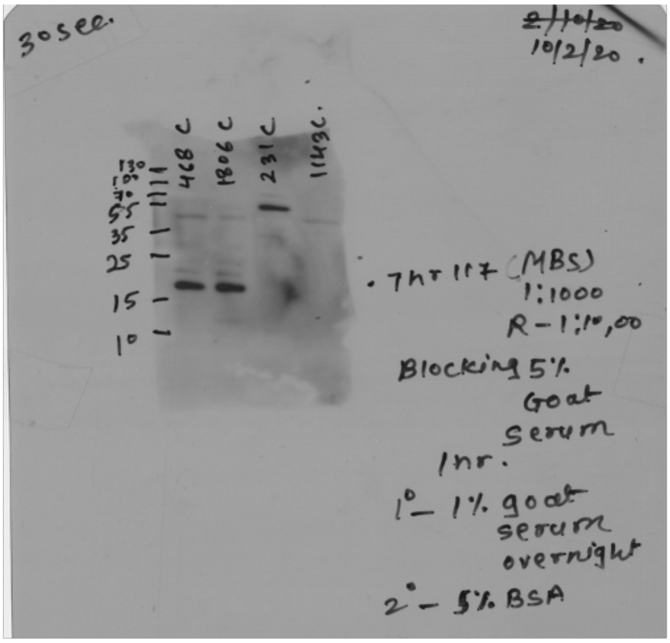

Figure-3A: PLK1

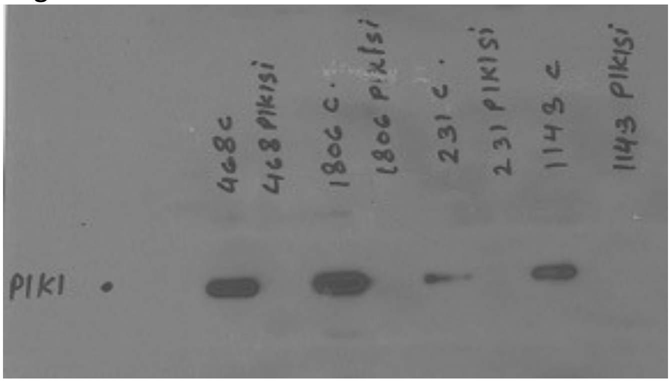

Figure-3A: Beta-actin

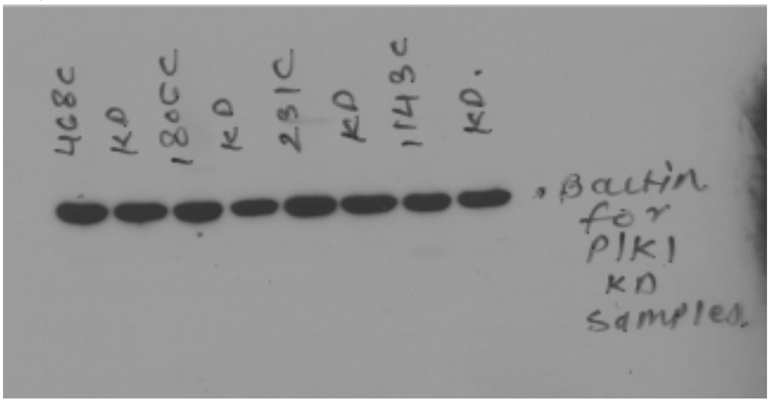

Figure-3A: Survivin S-20

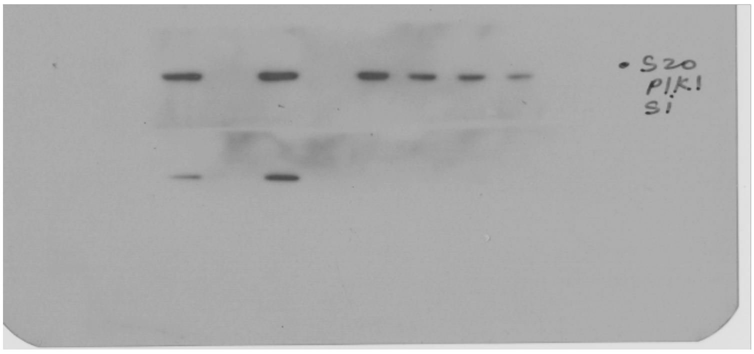

Figure-3A: Survivin

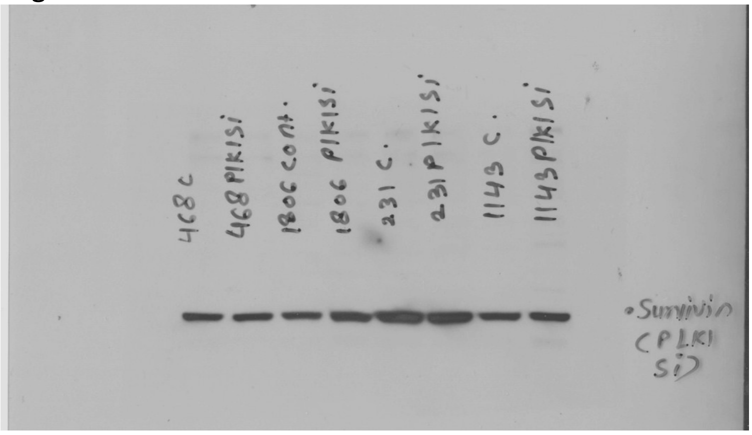

Figure-3B: PLK1 and Survivin T-117

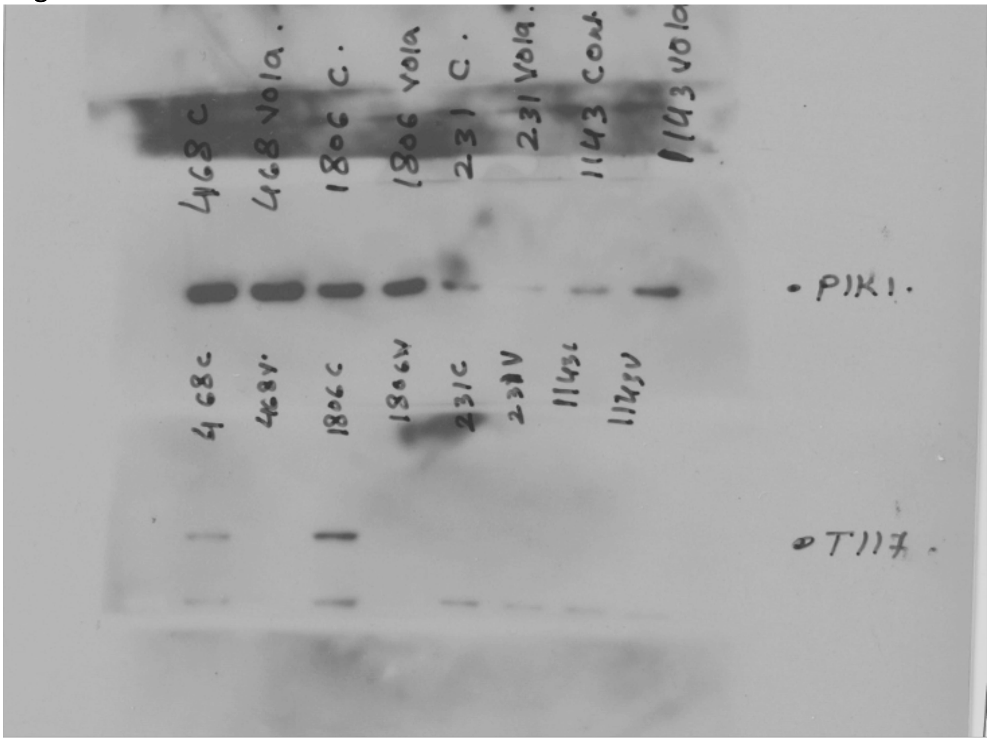

Figure-3B: Beta-actin and Survivin S-20

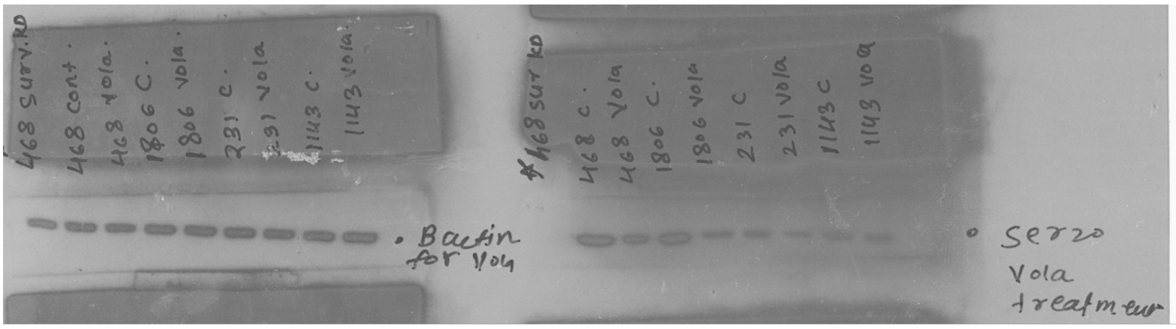

Figure-3B: Survivin

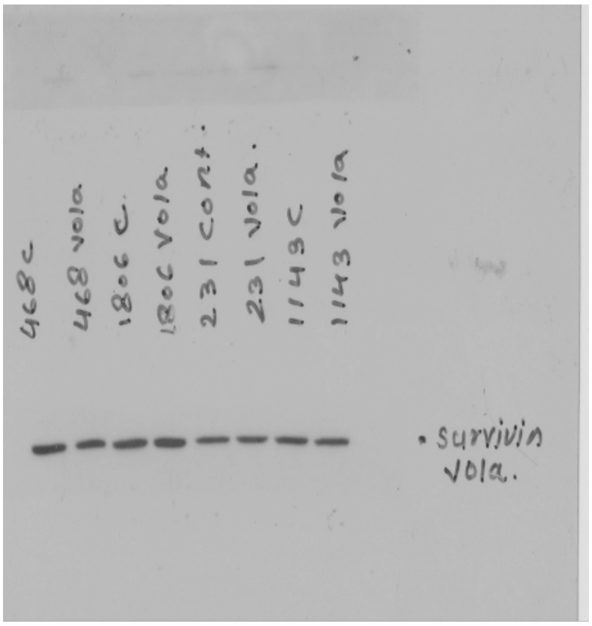

Figure-3C: AURKB

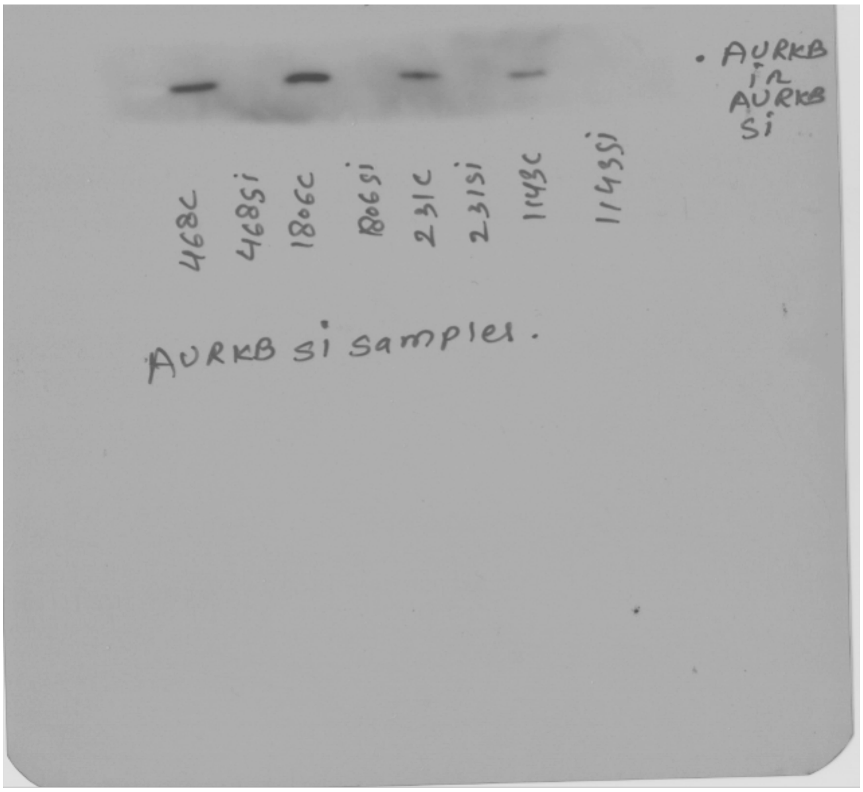

Figure-3C: Survivin

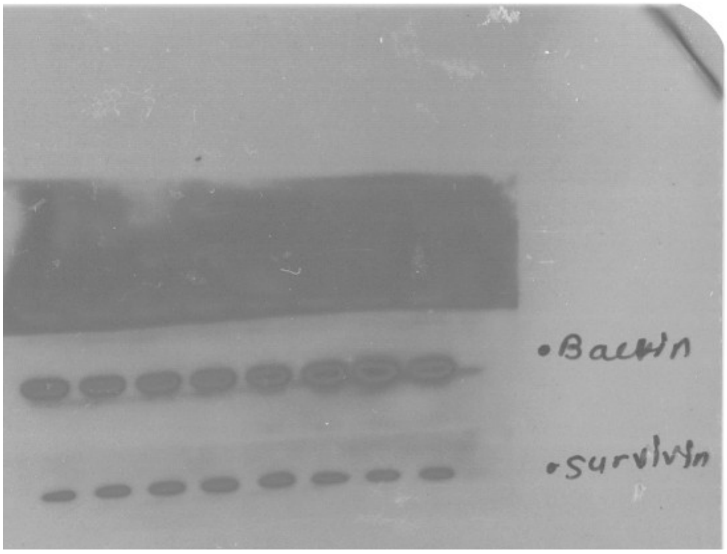

Figure-3C: Beta-actin

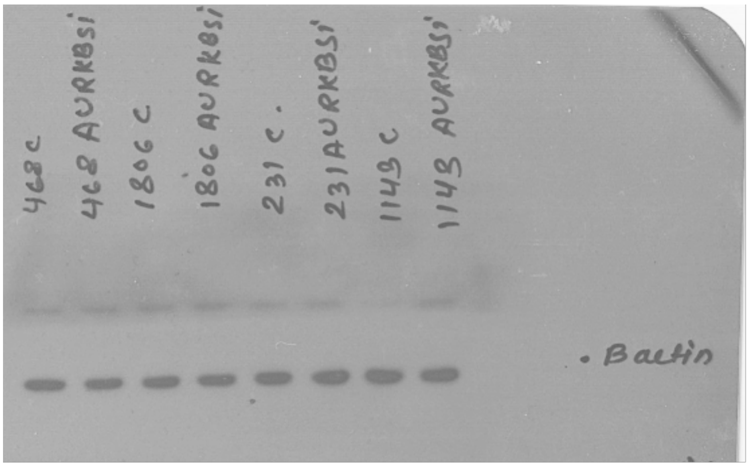

Figure-3C: Survivin T-117

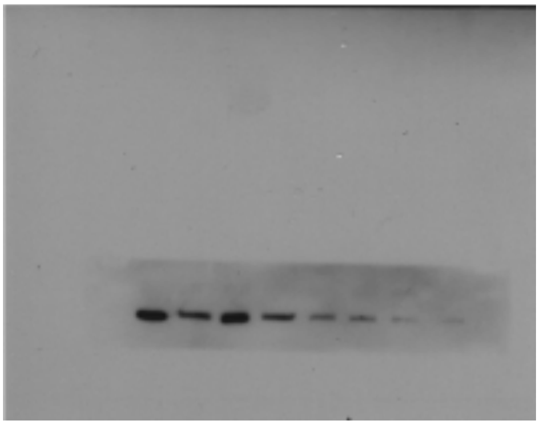

Figure-3D: AURKB

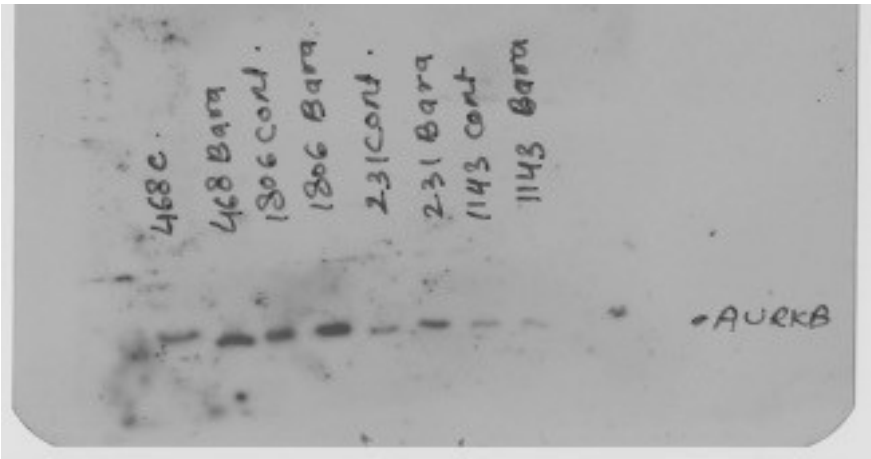

Figure-3D: Survivin

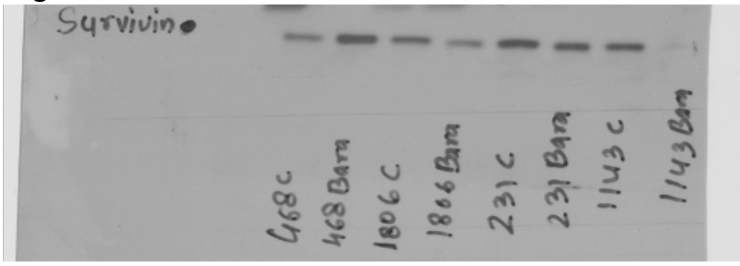

Figure-3D: Survivin S-20

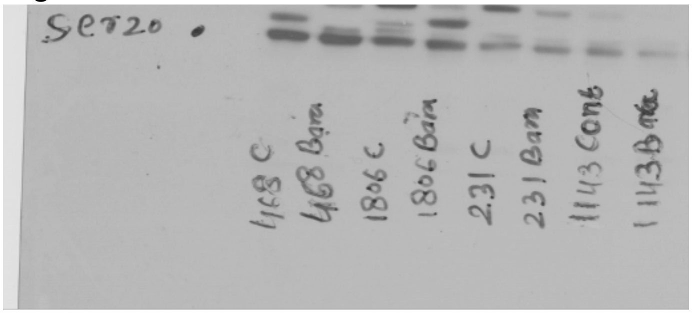

Figure-3D: Survivin T-117

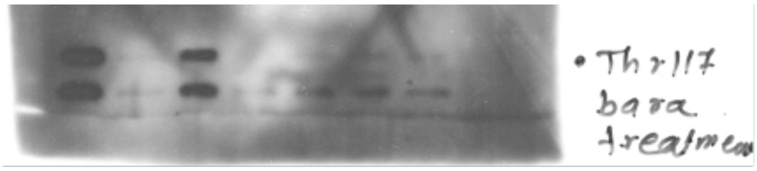

Figure-3D: Beta-actin

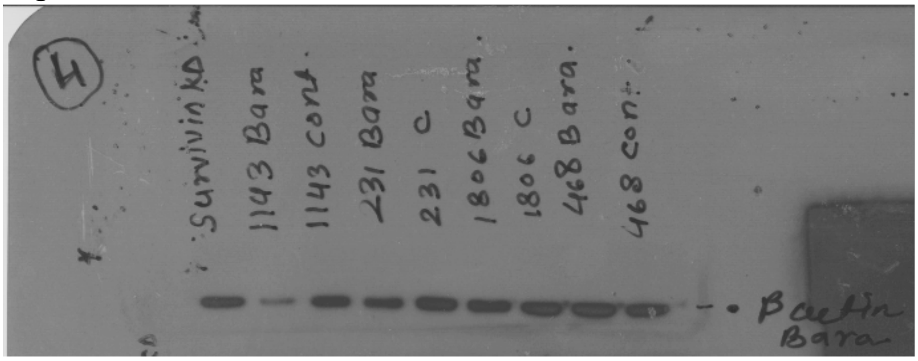

Figure-6G: AURKB

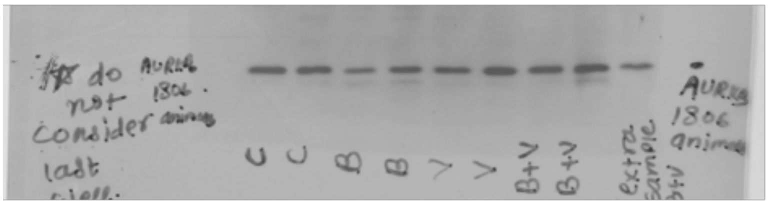

Figure-6G: PLk1

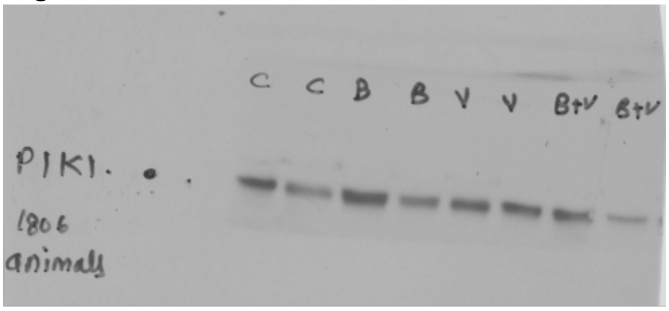

Figure-6G: Survivin

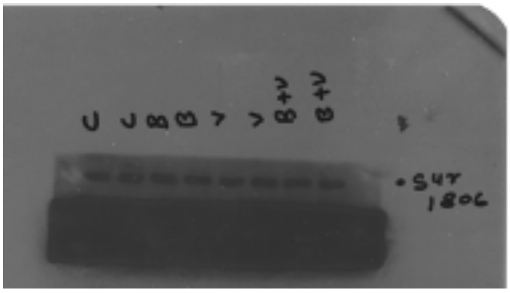

Figure-6G: Survivin S-20

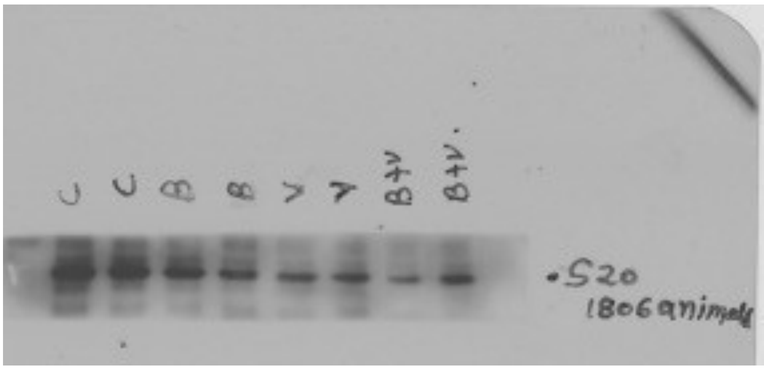

Figure-6G: Survivin T-117

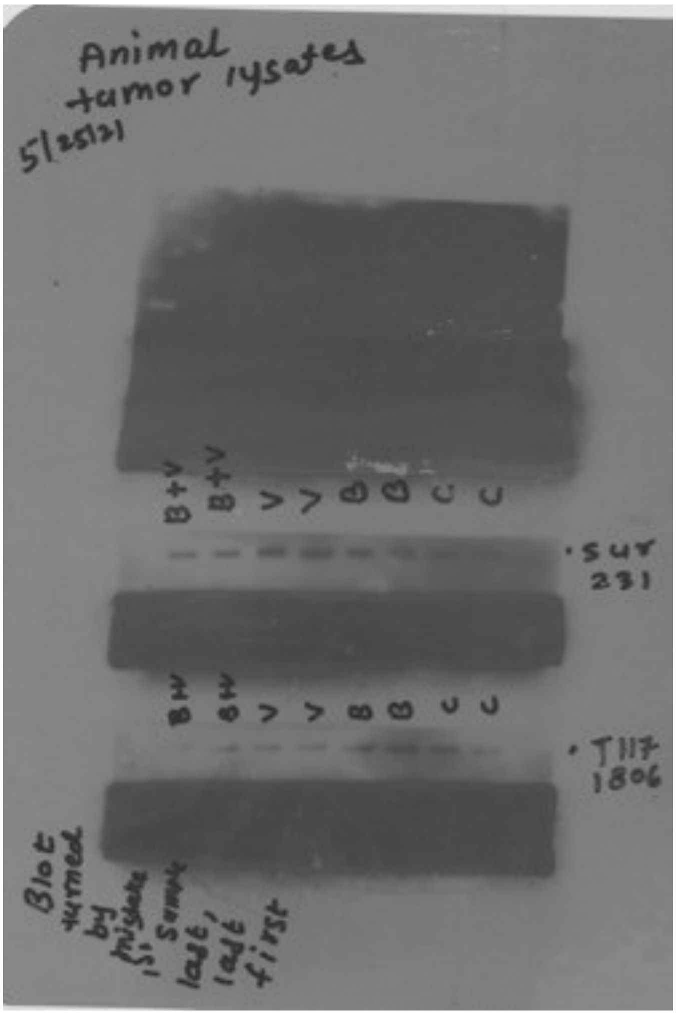

Figure-6G: Beta-actin

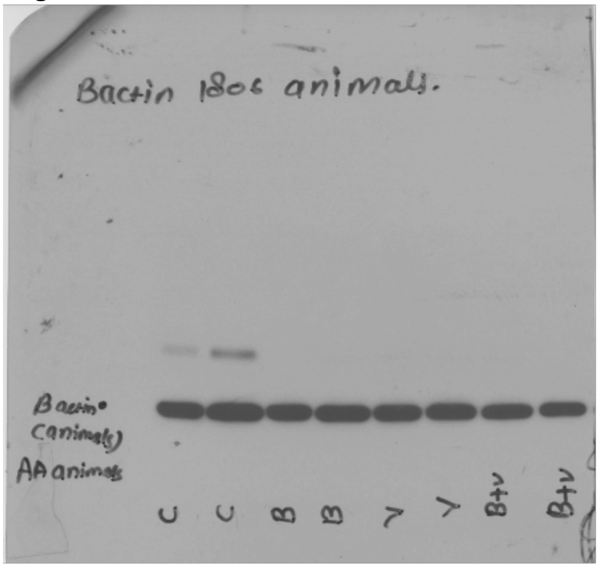

Figure-6H: AURKB

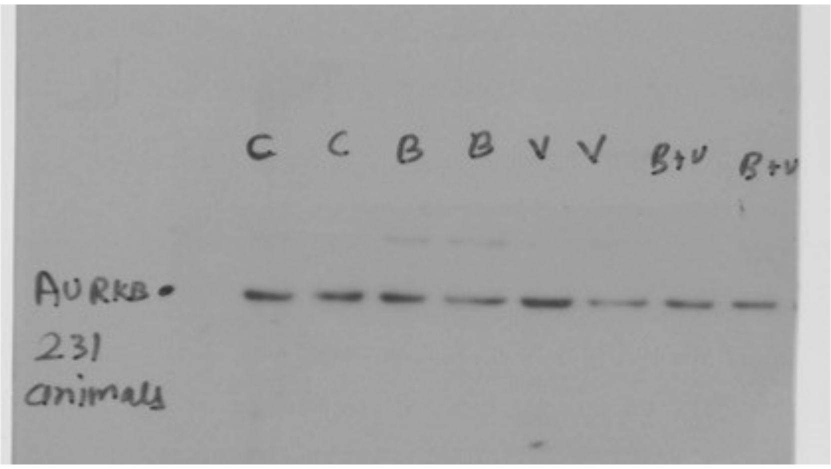

Figure-6H: PLK1

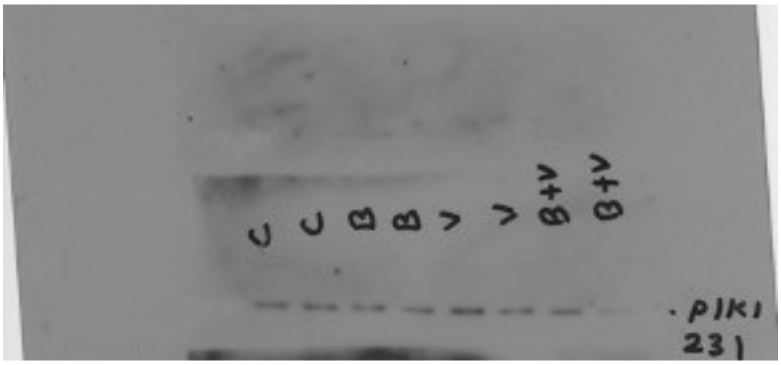

Figure-6H: Survivin

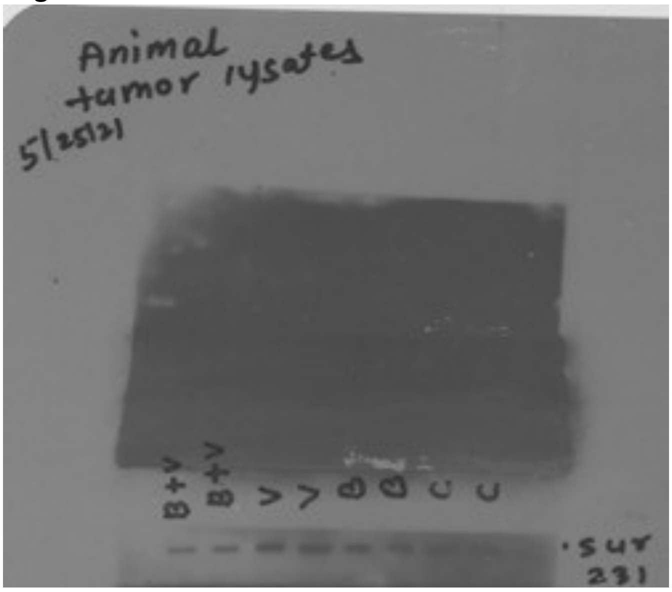

Figure-6H: Survivin S-20

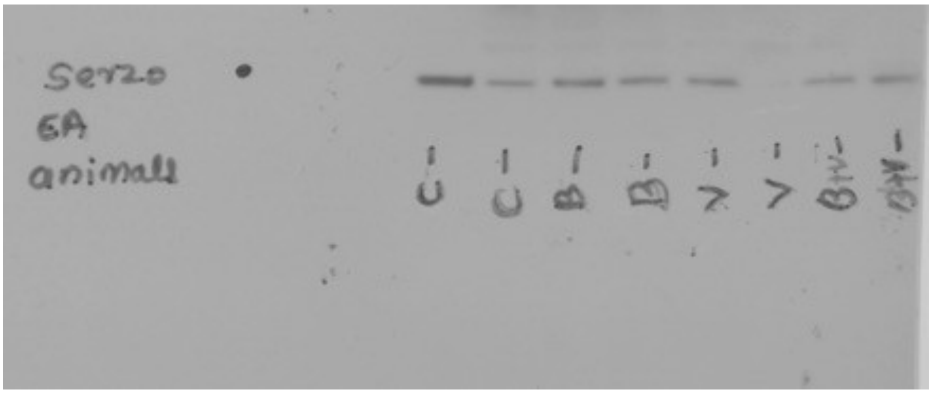

Figure-6H: Survivin T-117

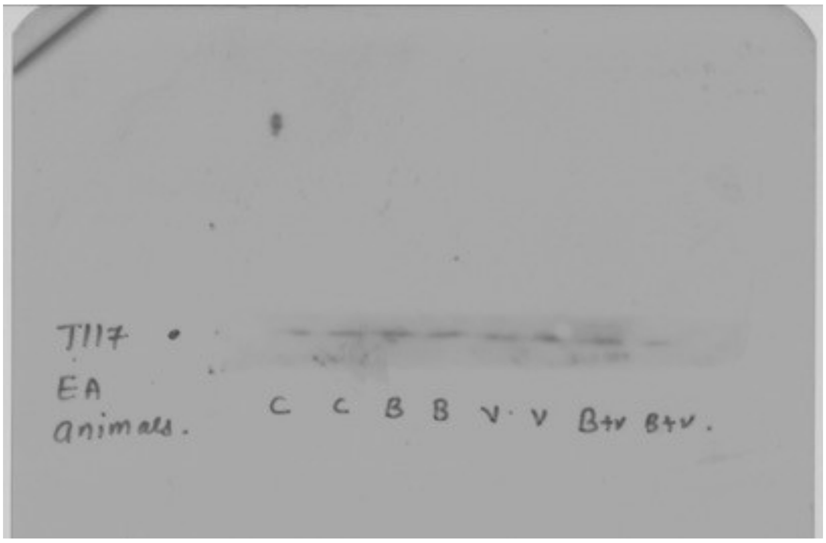

Figure-6H: Beta-actin

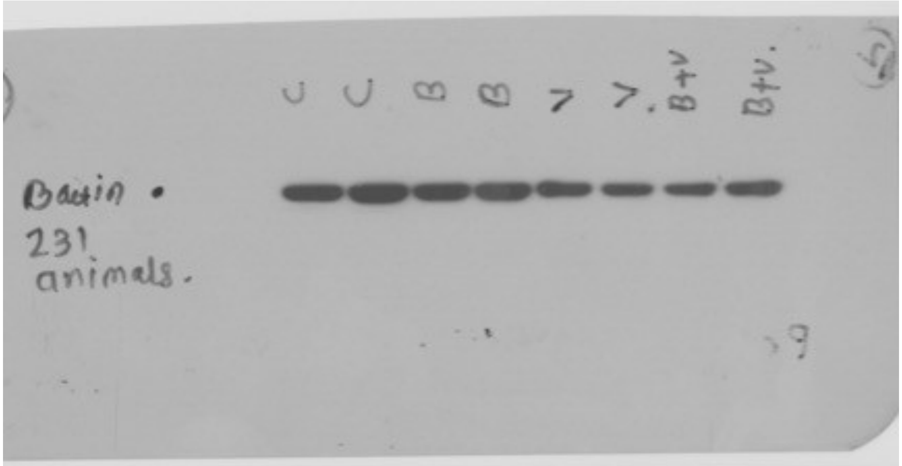

Figure-7B: Flag tag

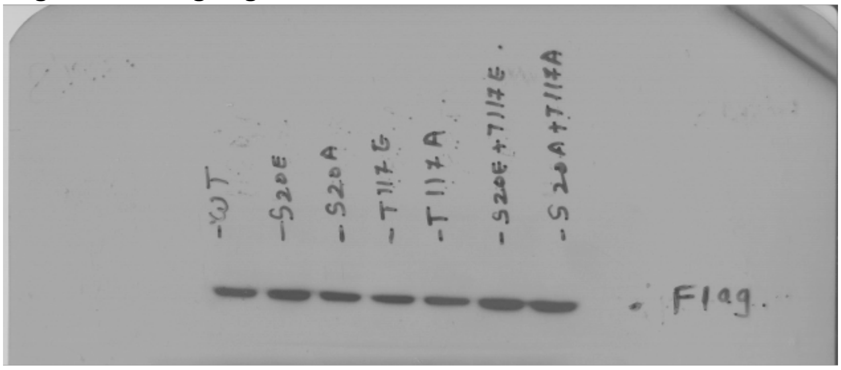

Figure-7B: INCENP

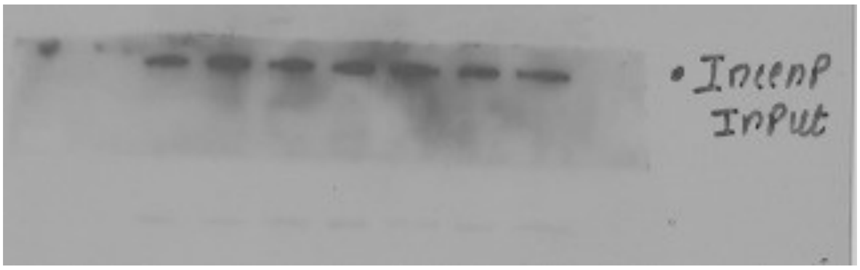

Figure-7B: Borealin

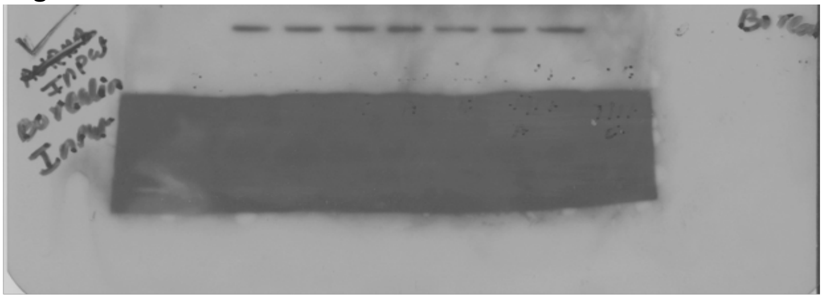

Figure-7B: AURKB

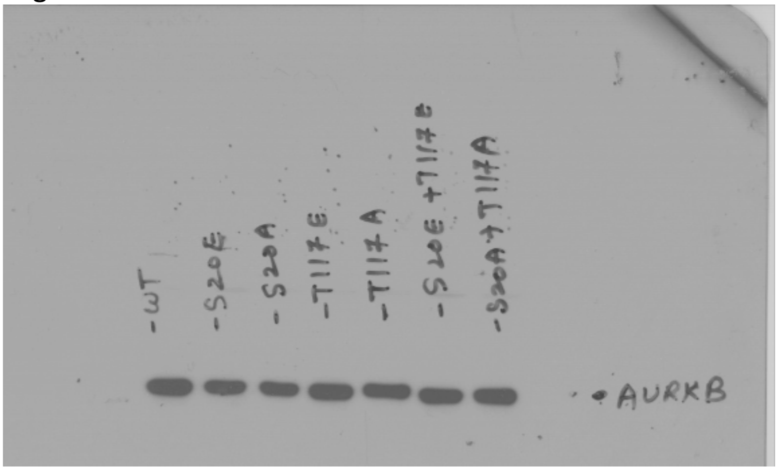

Figure-7B: Beta-actin

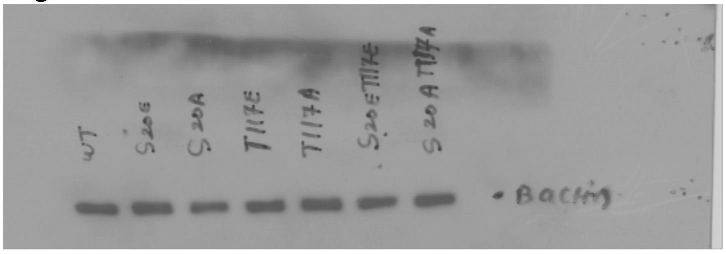

Figure-7C: Flag tag bound

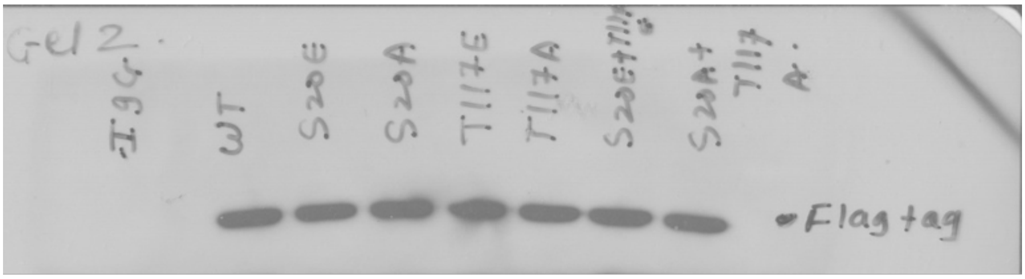

Figure-7C: Borealin bound protein

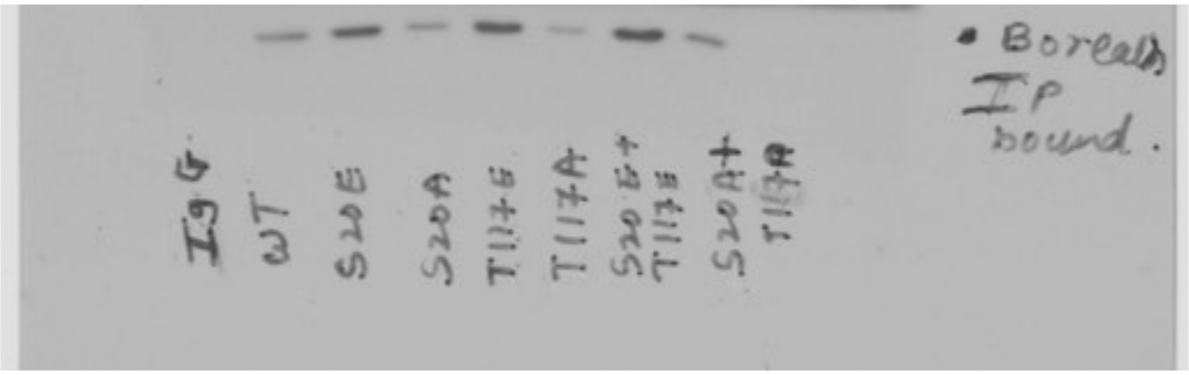

Figure-7C: Bound Survivin S-20

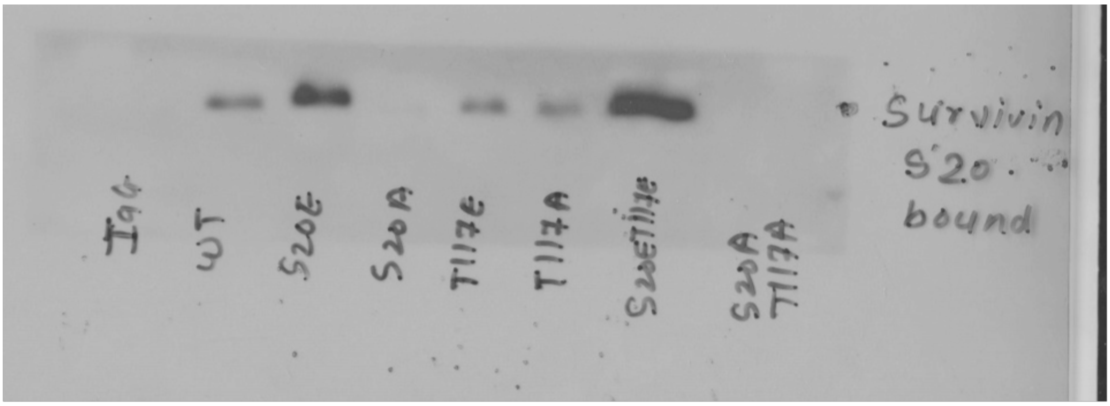

Figure-7C: Bound AURKB

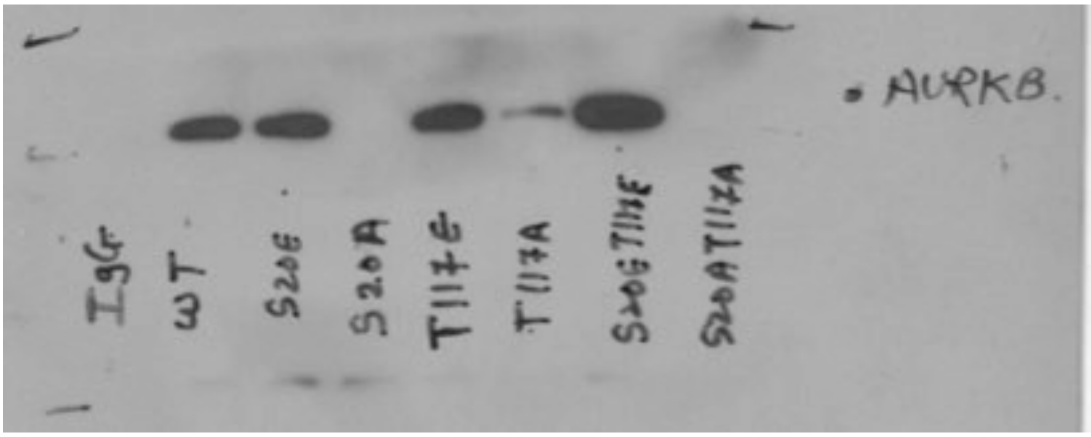

Figure-7C: Survivin T-117 bound protein

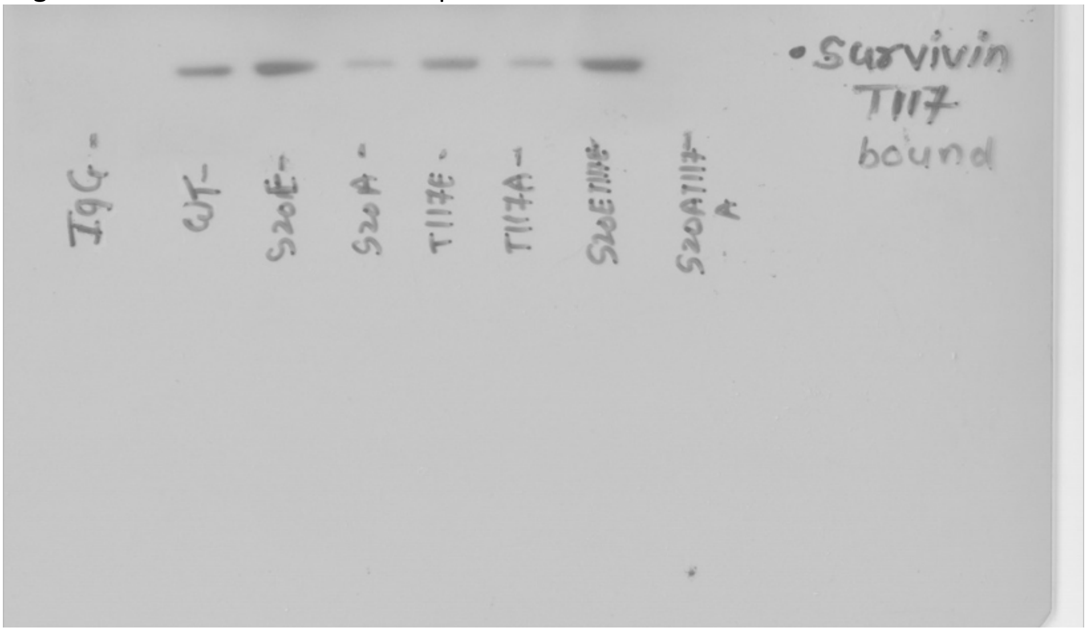

Figure-7C: INCENP

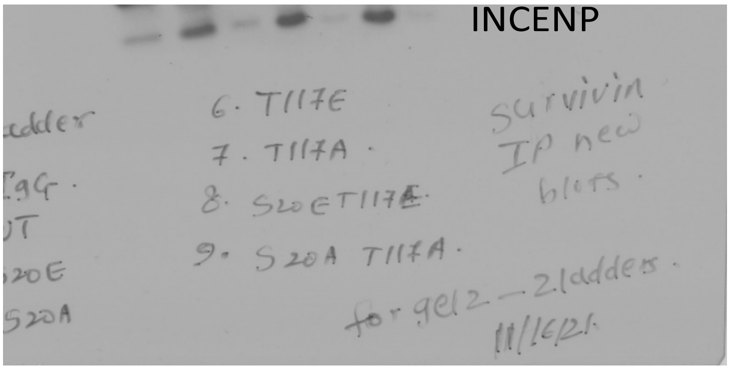

Supplementary figure-4A: Survivin and beta-actin of KD sampels

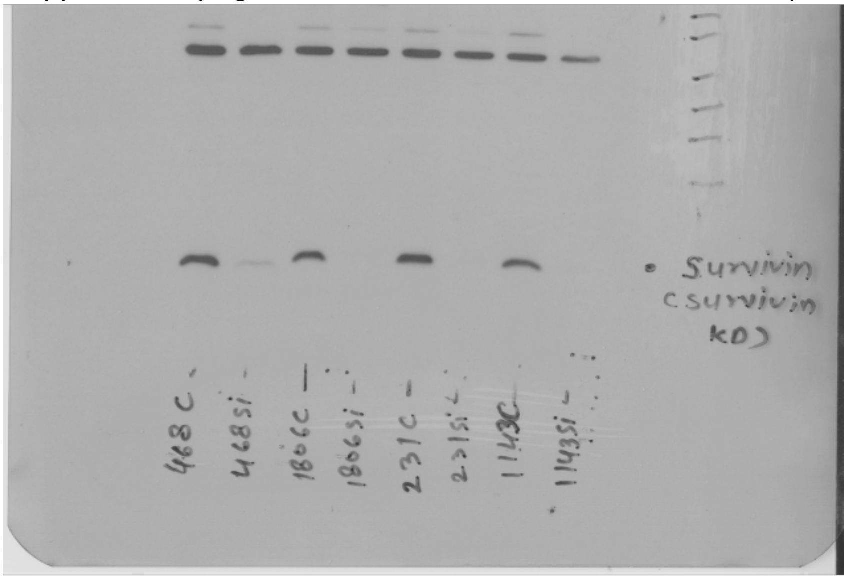

New raw blots after revision

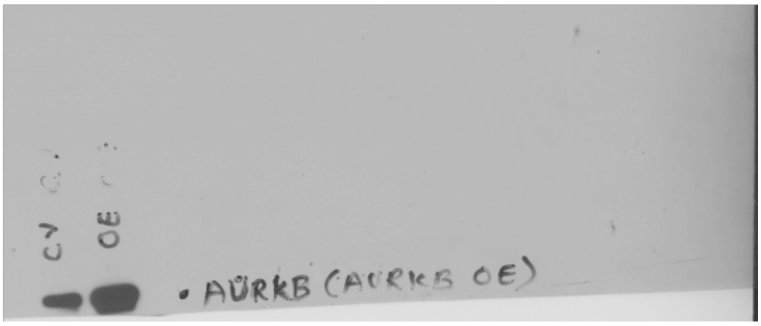

Supplementary figure-8A: PLK1 OE stable cell line

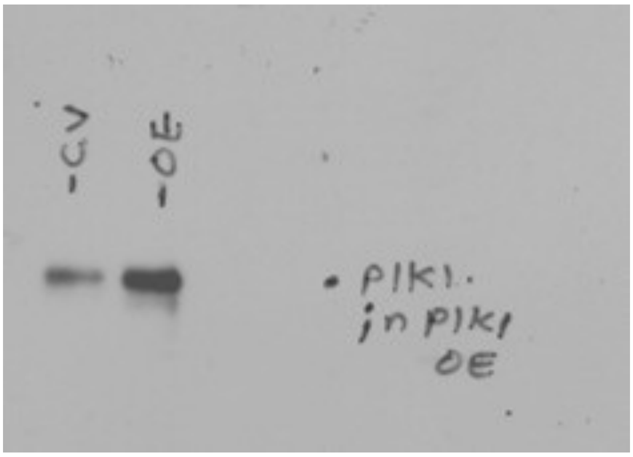

Supplementary figure-8A: Beta-actin

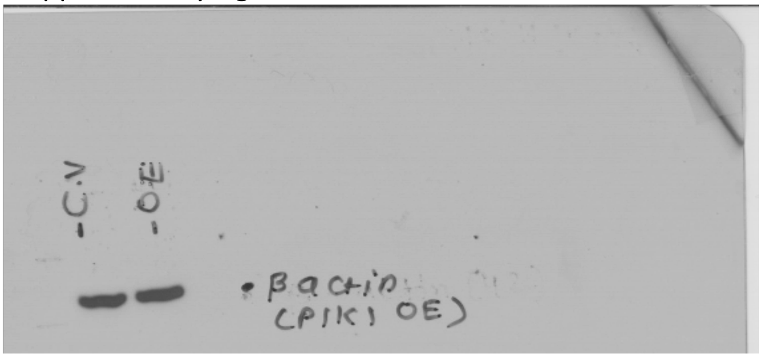

New raw blots after revision

Main Figure 1 I, PLK1, AURKB, beta actin basal level 3 AA and 3 EA TNBC cells

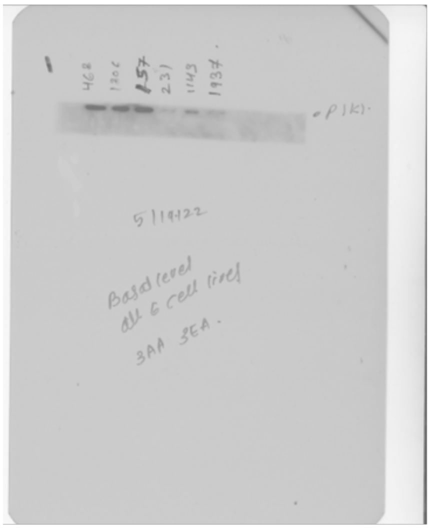

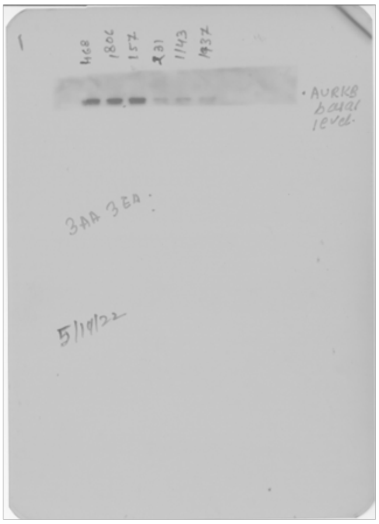

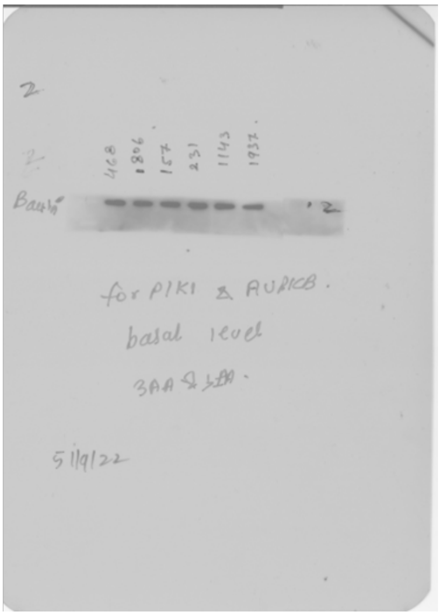

Main Figure 2 D, Survivin and beta actin basal level 3 AA and 3 EA cell lines

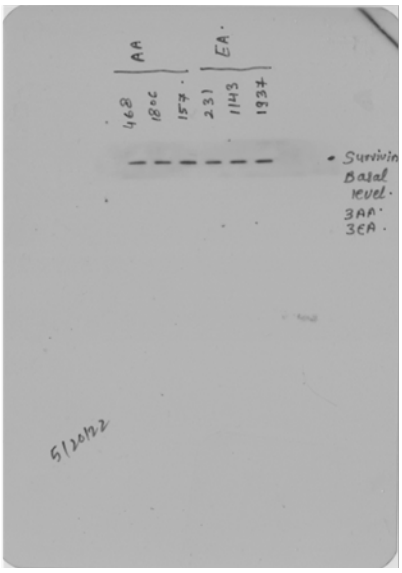

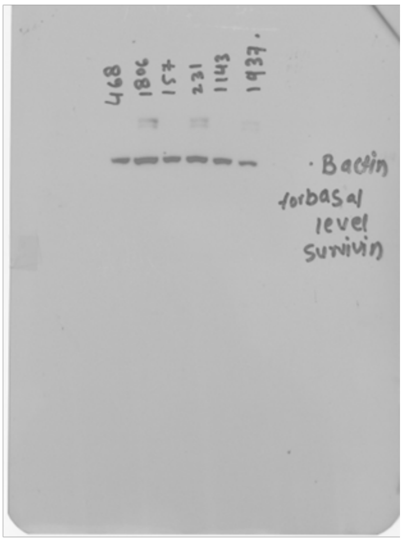

New raw blots after revision

Main figure 2I, S20, T117, Beta actin basal level 3 AA and 3 EA cell lines

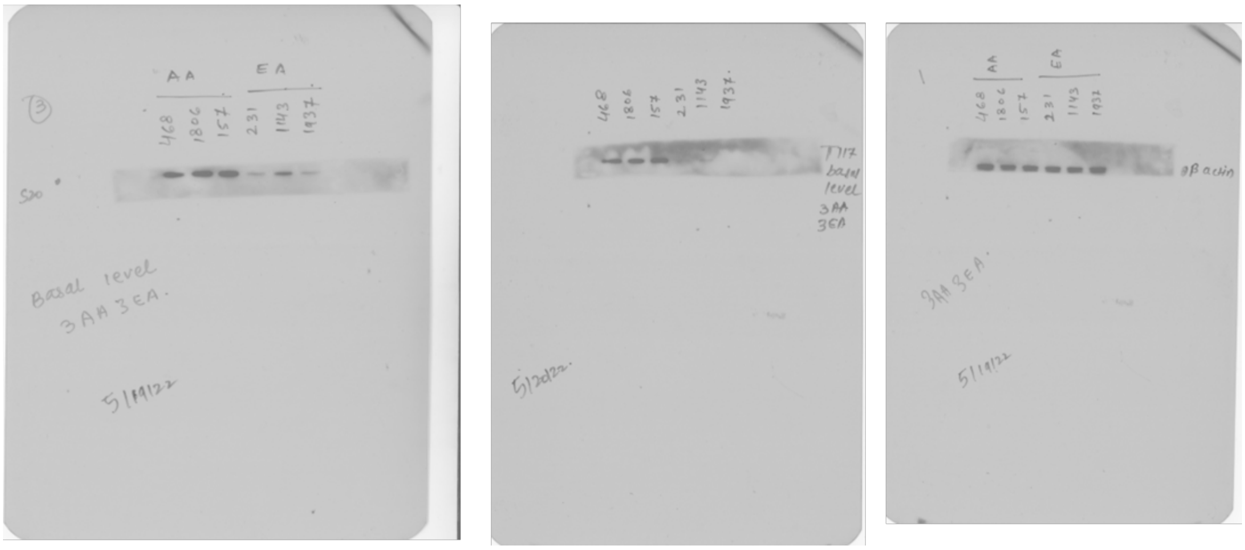

Main Figure 3 B, E New set of PLK1 and AURKB siRNA (PLK1 siRNA 2, AURKB si RNA 2) in 2 AA and 2 EA cell lines

Figure 3B PLK1, Survivin, S20, beta actin in PLK1 SiRNA 2

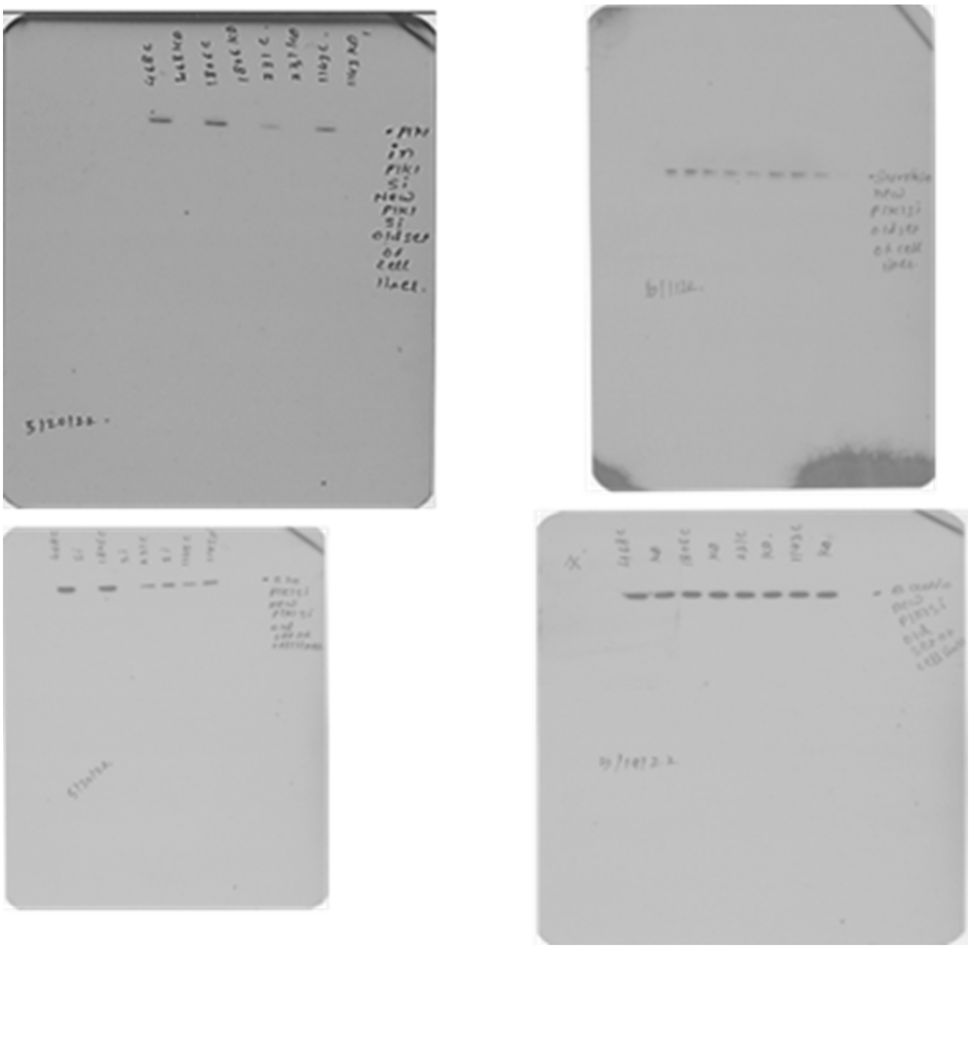

New raw blots after revision

Figure 3 E AURKB, Survivin, T117, beta actin in AURKB SiRNA 2

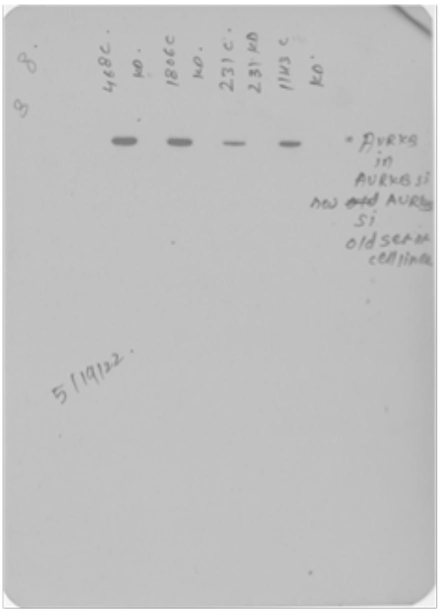

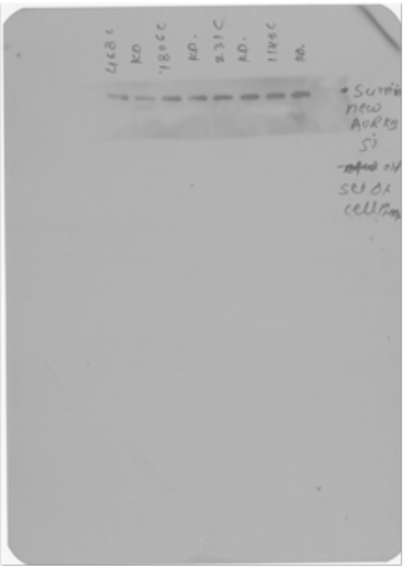

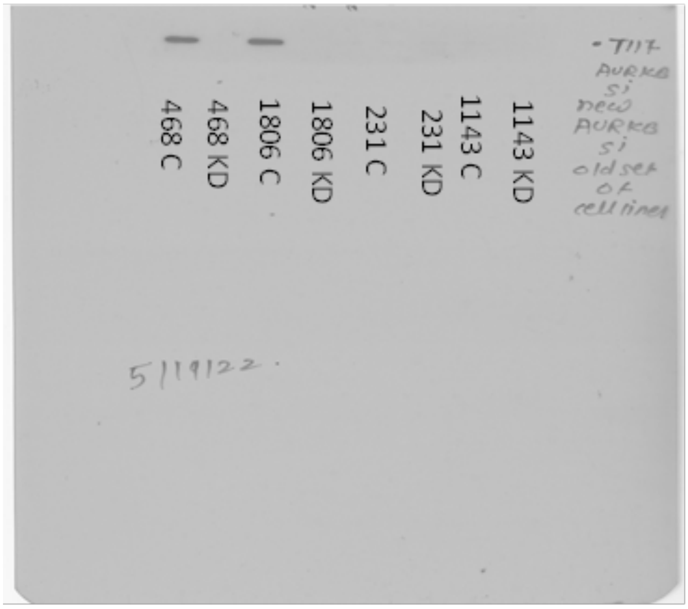

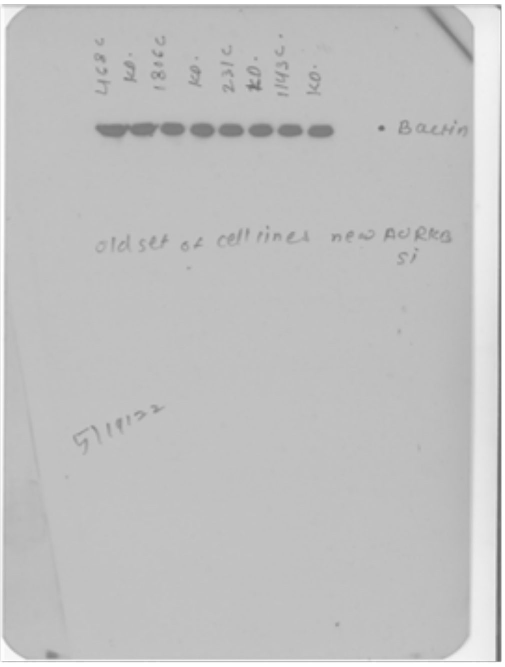

New raw blots after revision

Supplementary figures
Supplementary figure 2 C-G
Supp. Fig 2 C PLK1 siRNA 1 new set of cell lines 1 AA, 1 EA

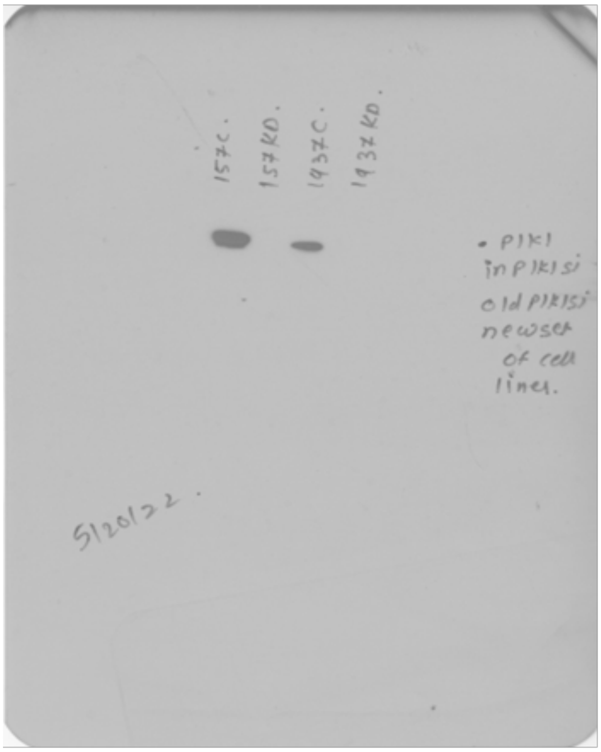

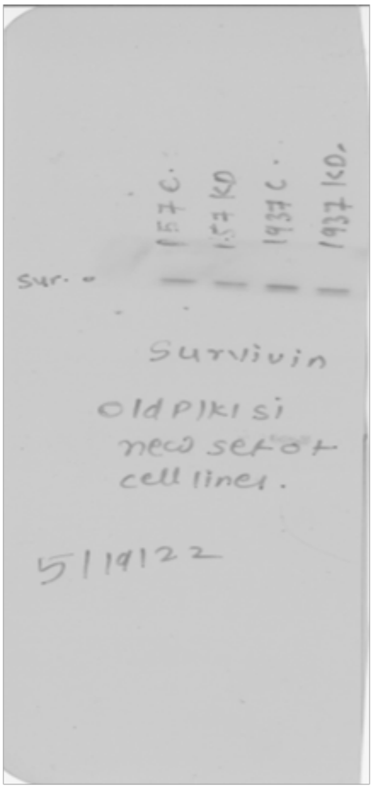

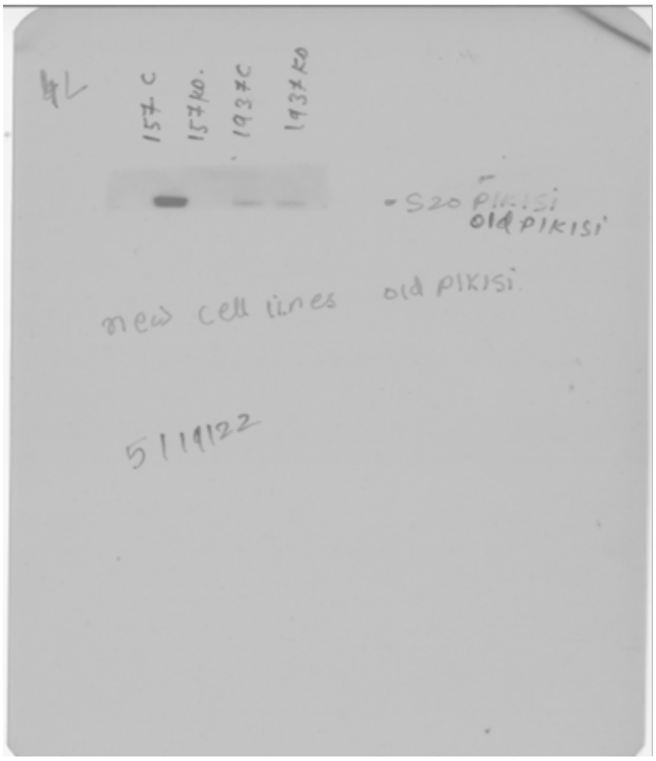

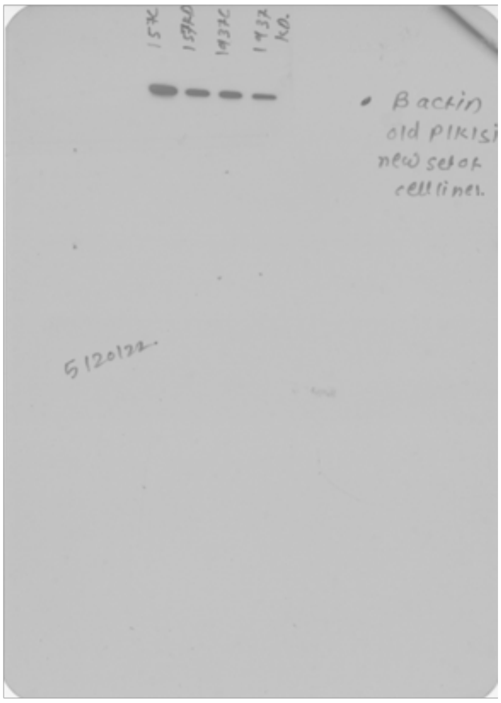

New raw blots after revision

Supp. Fig 2 D PLK1 siRNA 2 new set of cell lines 1 AA, 1 EA

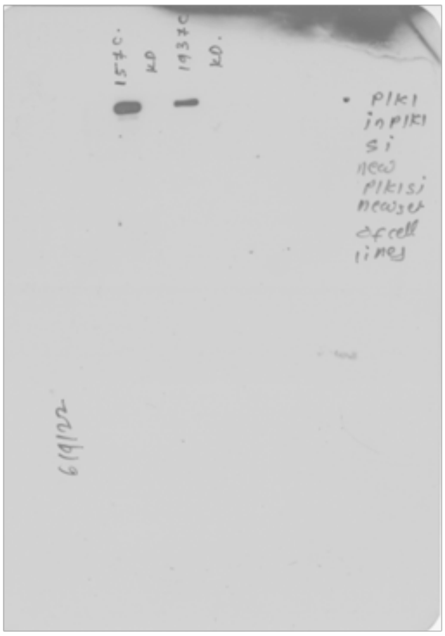

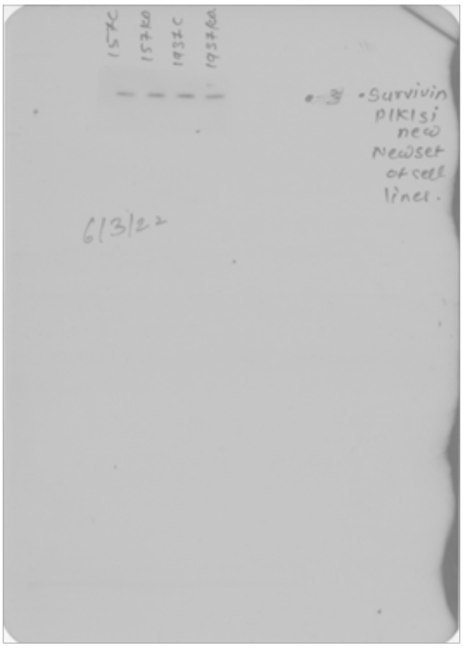

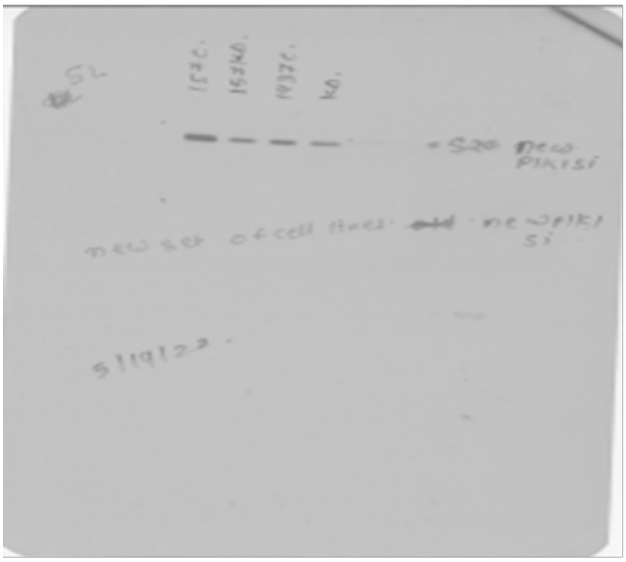

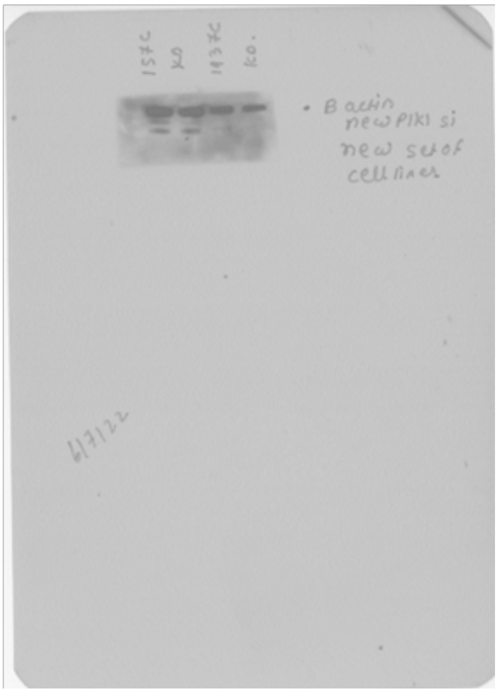

New raw blots after revision

Supp. Fig 2 E AURKB siRNA 1/2 new set of cell lines 1 AA, 1 EA

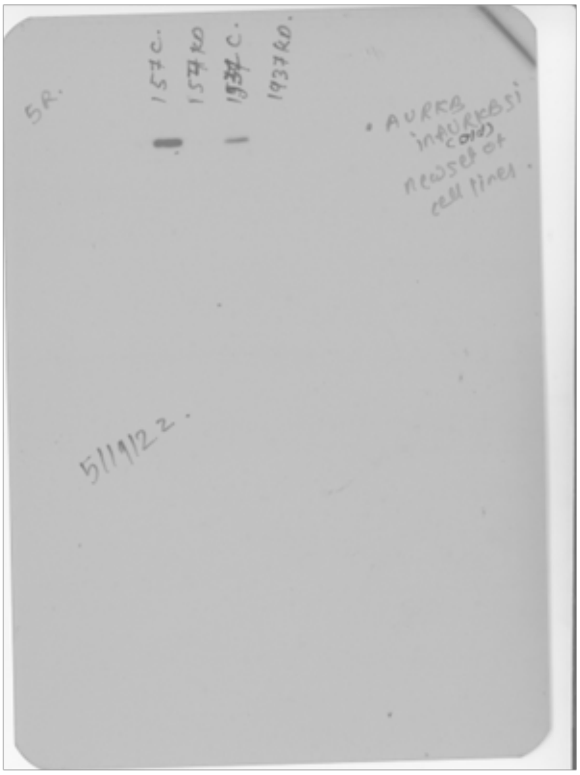

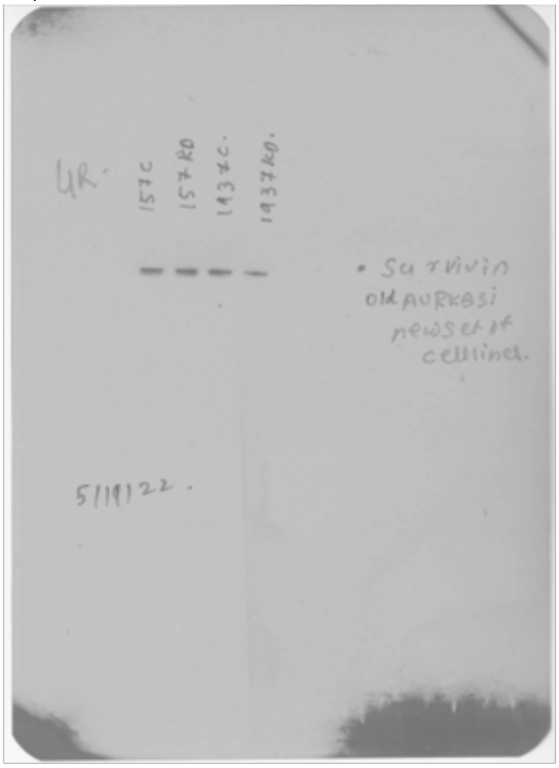

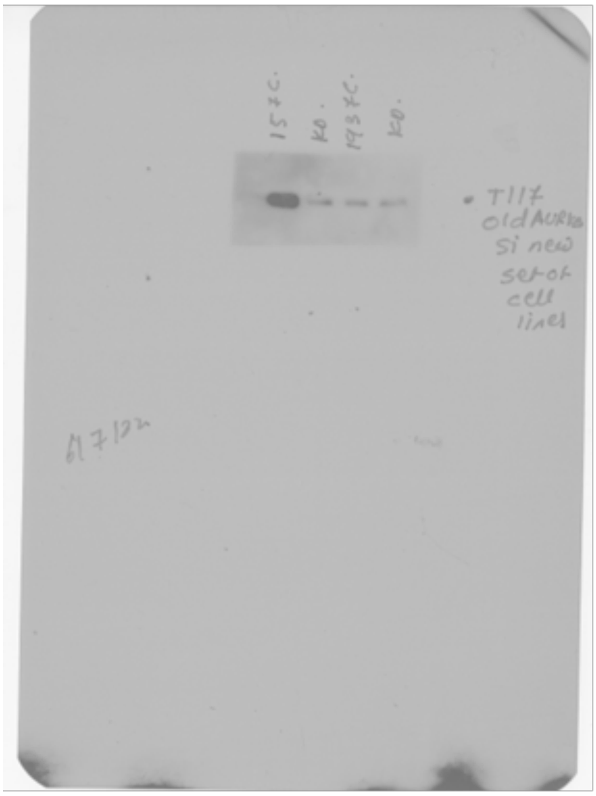

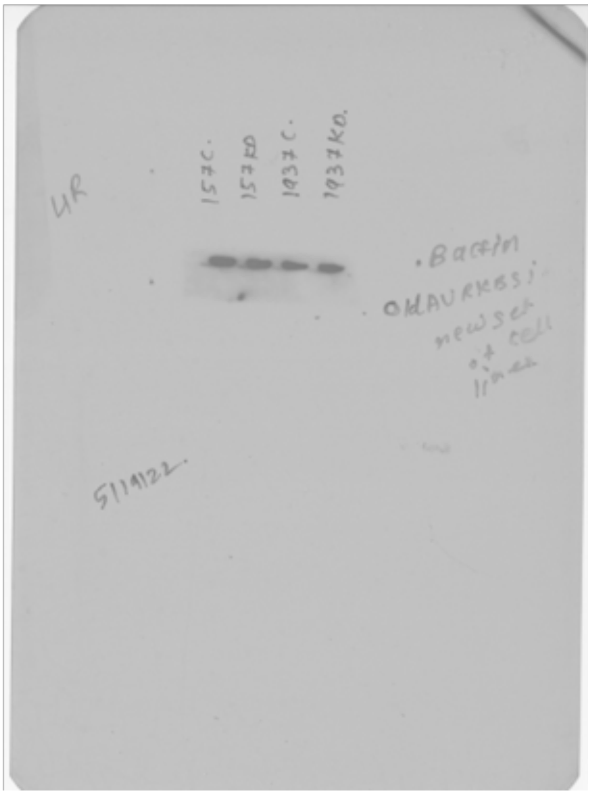

New raw blots after revision

Supp. Fig 2 F Volasertib new set of cell lines 1 AA, 1 EA, PLK1, Survivin, S20, T117, B actin

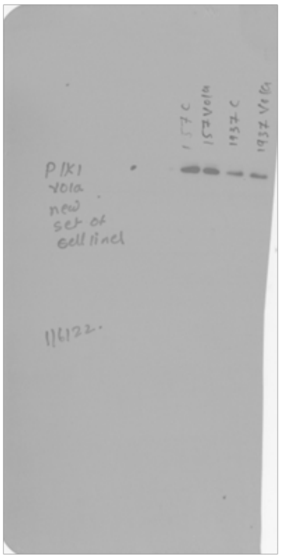

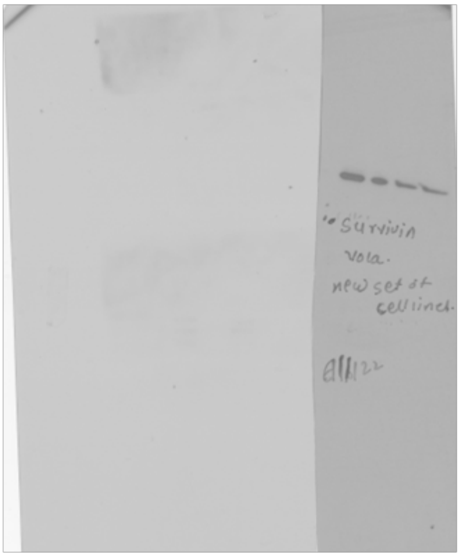

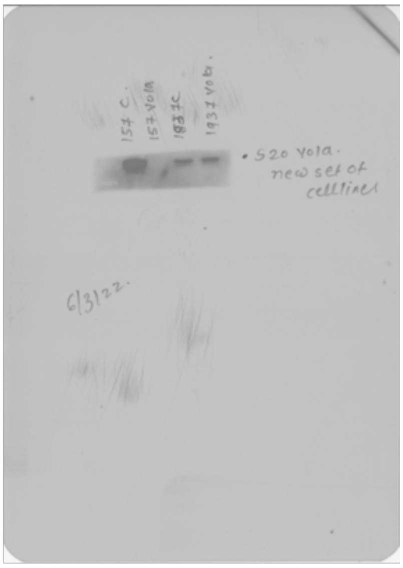

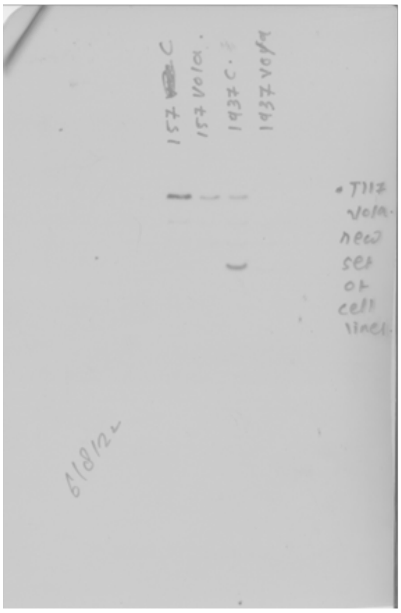

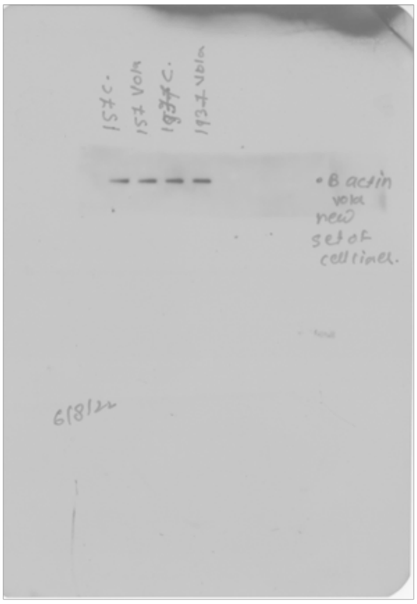

New raw blots after revision

Supp. Fig 2 G Barasertib new set of cell lines 1 AA, 1 EA, AURKB, Survivin, S20, T117, B actin

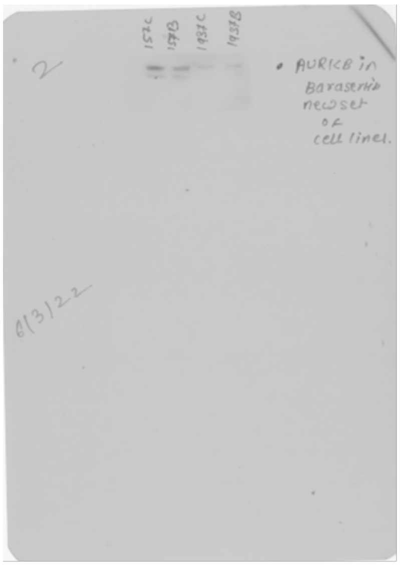

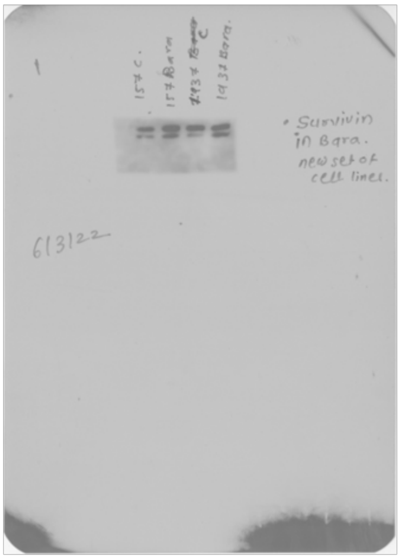

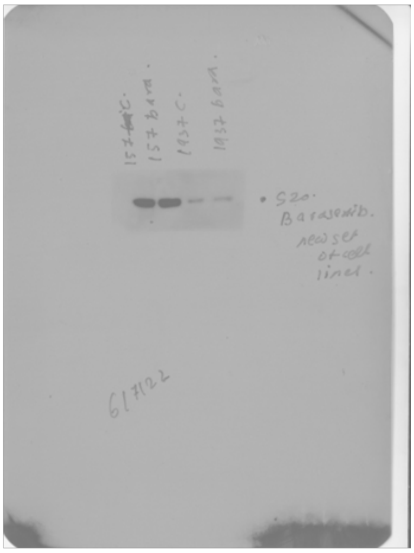

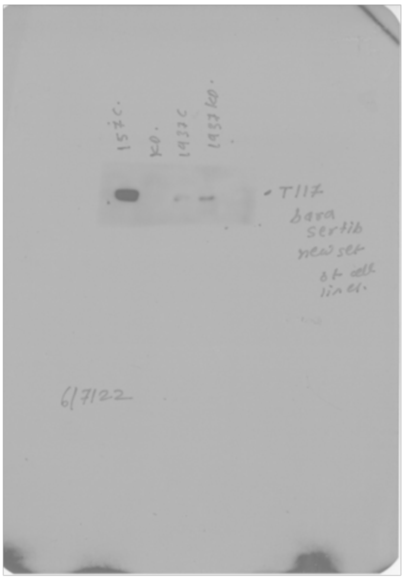

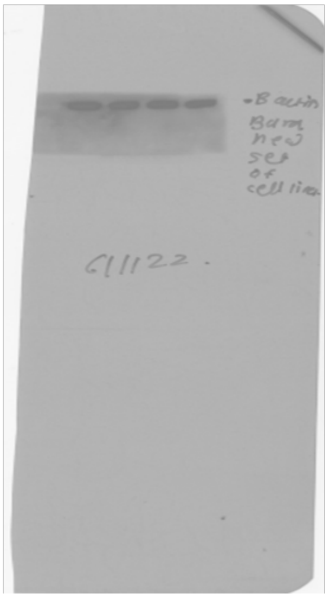

New raw blots after revision

Revision 11.01.22

Suppl. Figure 3A-Immunoblots showing the levels of total survivin and p-survivin (S20, T117) upon PLK1 overexpression

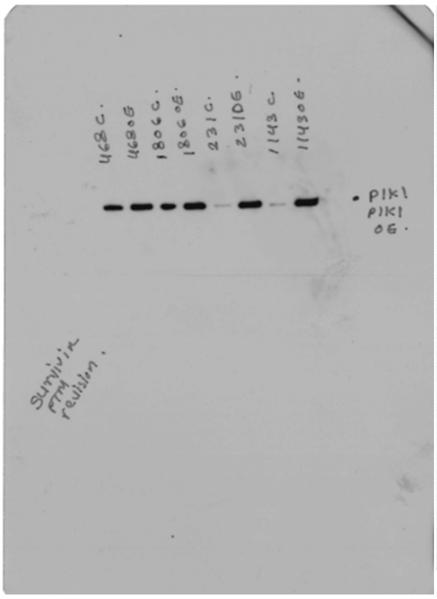

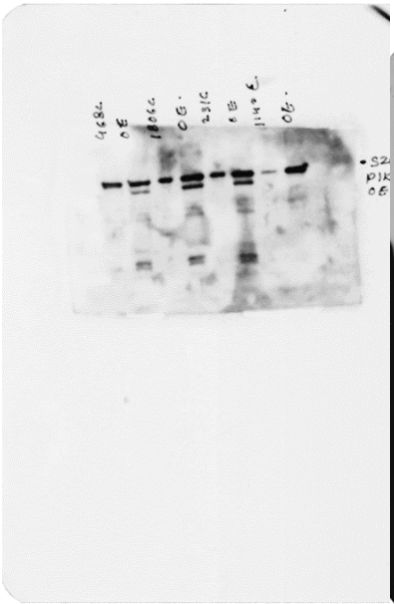

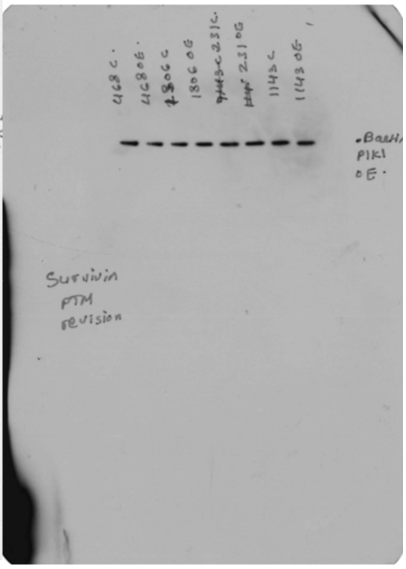

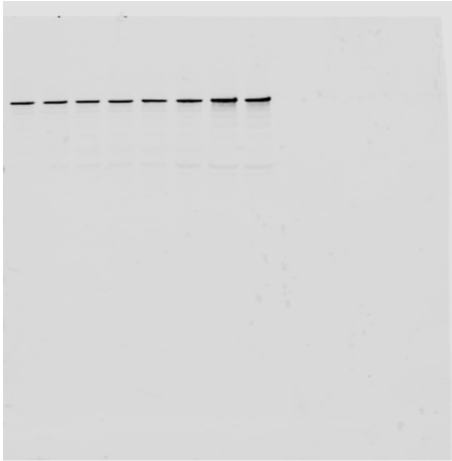

New raw blots after revision

Suppl. Figure 3B-Immunoblots showing the levels of total survivin and p-survivin (S20) upon PLK1 overexpression

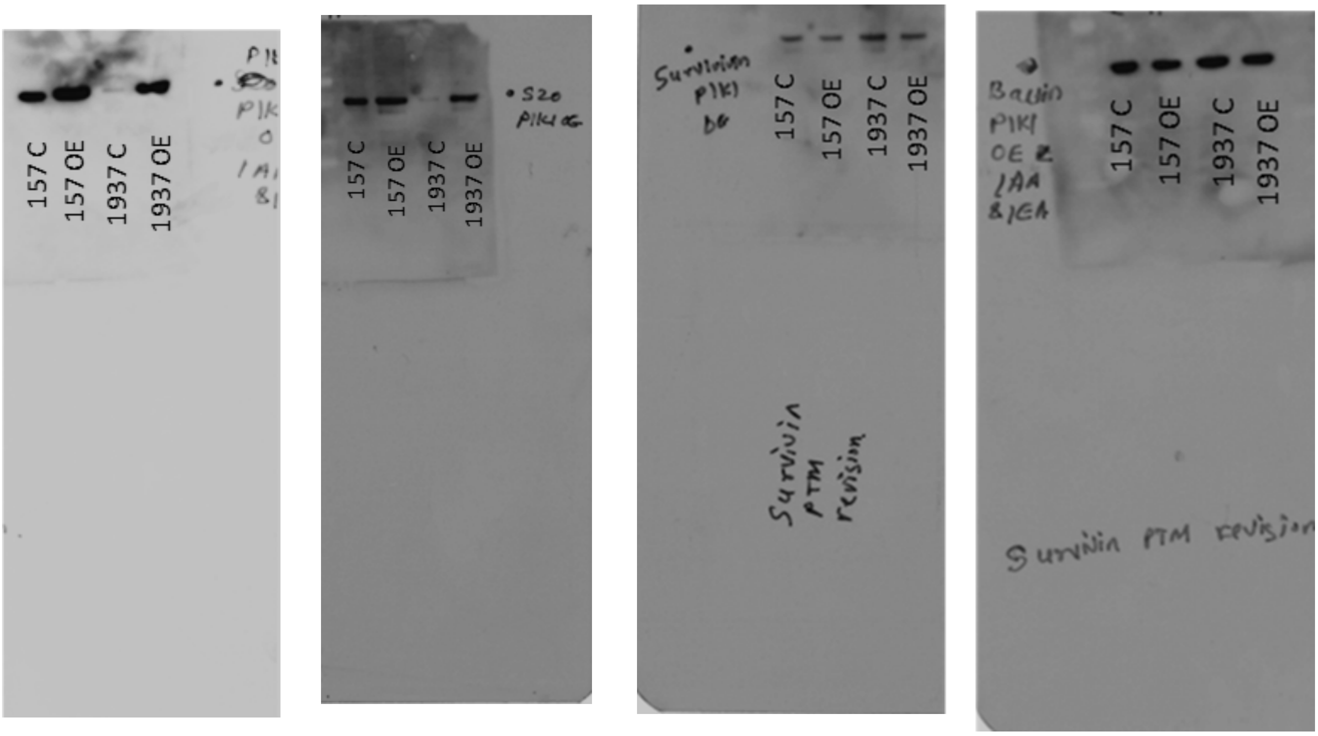

Suppl. Figure 3C- Immunoblots showing the levels of total survivin and p-survivin (T117) upon AURKB overexpression

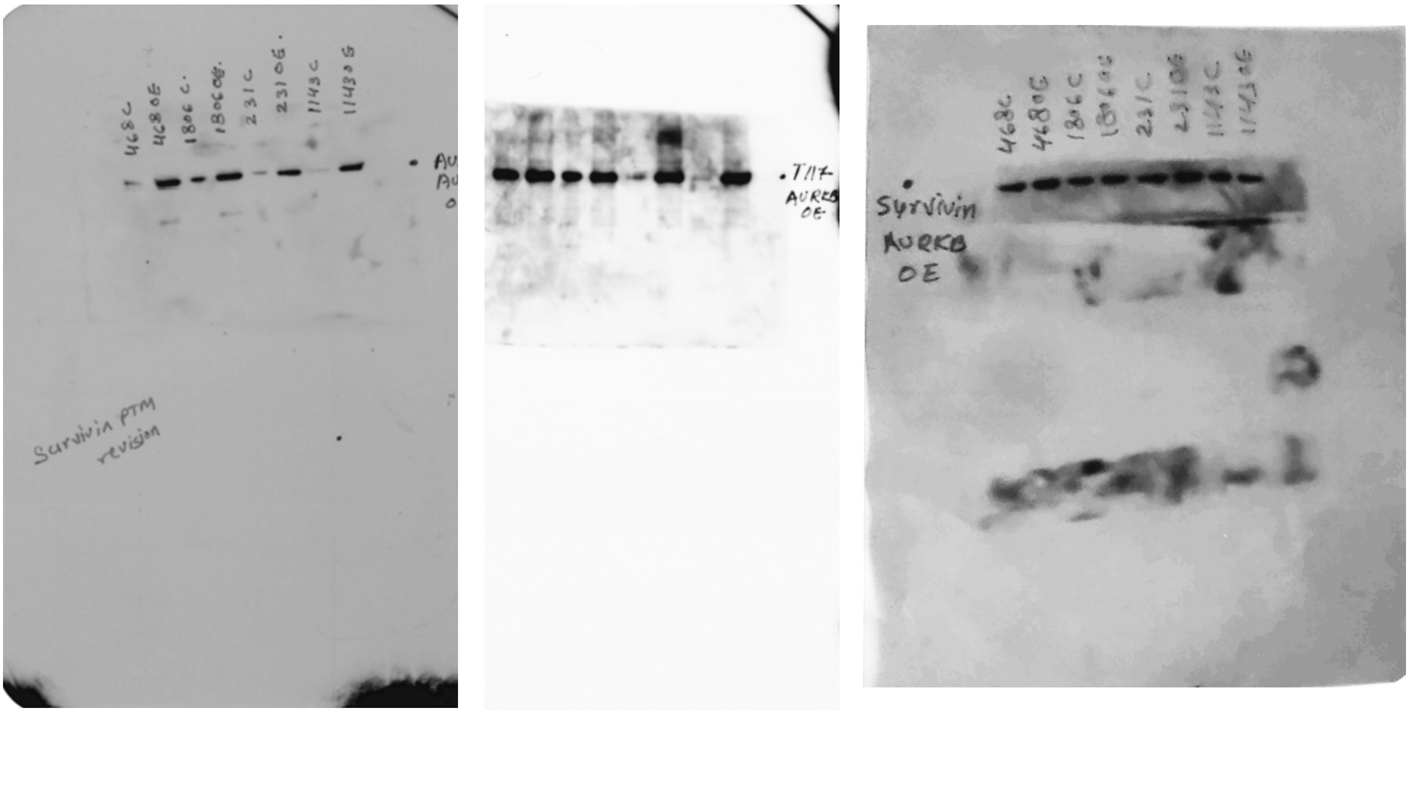

New raw blots after revision

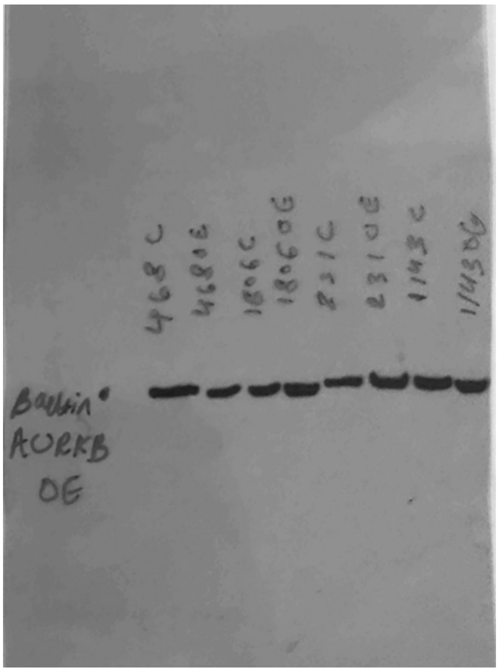

Suppl. Figure 3D- Immunoblots showing the levels of total survivin and p-survivin (T117) upon AURKB overexpression

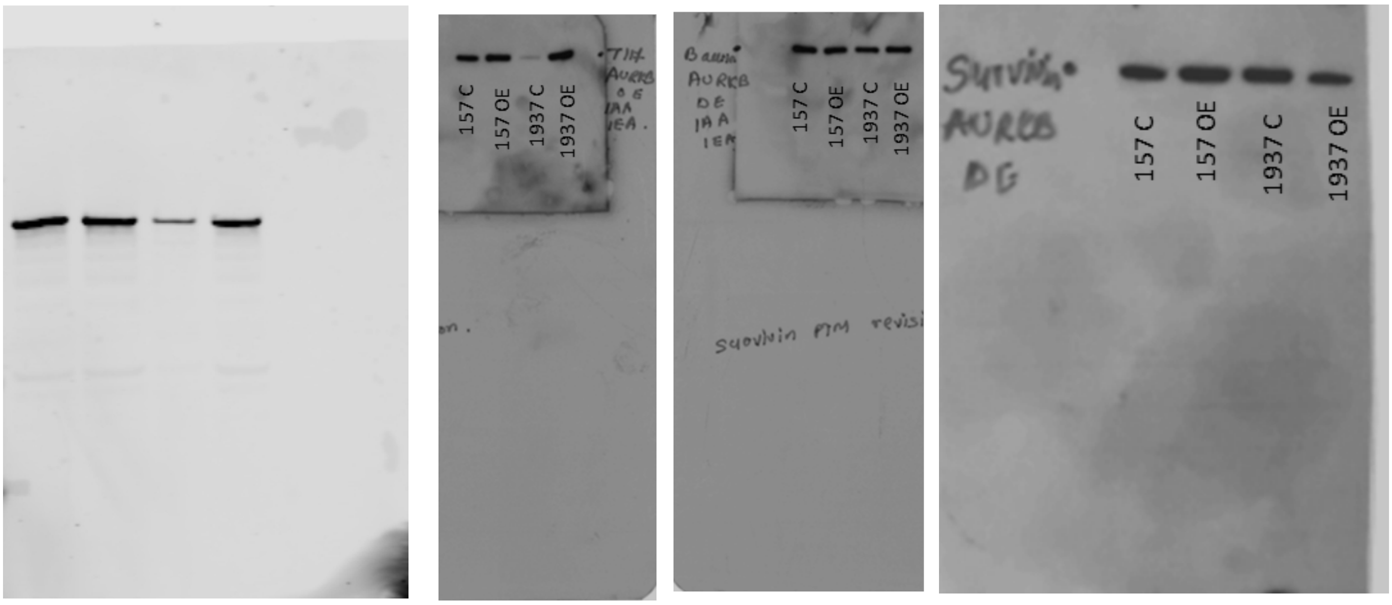

Supplement: Supplementary file 2 — All raw WB old and revised [file 41419_2022_5539_MOESM2_ESM.pdf]
